# Global, regional, and national burden of gout, 1990–2020, and projections to 2050: a systematic analysis of the Global Burden of Disease Study 2021

**DOI:** 10.1016/S2665-9913(24)00117-6

**Published:** 2024-07-09

**Authors:** GBD 2021 Gout Collaborators, Marita Cross, Kanyin Liane Ong, Garland T Culbreth, Jaimie D Steinmetz, Ewerton Cousin, Hailey Lenox, Jacek A Kopec, Lydia M Haile, Peter M Brooks, Deborah R Kopansky-Giles, Karsten E Dreinhoefer, Neil Betteridge, Mohammadreza Abbasian, Mitra Abbasifard, Aidin Abedi, Melka Biratu Aboye, Aleksandr Y Aravkin, Al Artaman, Maciej Banach, Isabela M Bensenor, Akshaya Srikanth Bhagavathula, Ajay Nagesh Bhat, Saeid Bitaraf, Rachelle Buchbinder, Katrin Burkart, Dinh-Toi Chu, Sheng-Chia Chung, Omid Dadras, Xiaochen Dai, Saswati Das, Sameer Dhingra, Thanh Chi Do, Hisham Atan Edinur, Ali Fatehizadeh, Getahun Fetensa, Marisa Freitas, Balasankar Ganesan, Ali Gholami, Tiffany K Gill, Mahaveer Golechha, Pouya Goleij, Nima Hafezi-Nejad, Samer Hamidi, Simon I Hay, Samuel Hundessa, Hiroyasu Iso, Shubha Jayaram, Vidya Kadashetti, Ibraheem M Karaye, Ejaz Ahmad Khan, Moien AB Khan, Moawiah Mohammad Khatatbeh, Ali Kiadaliri, Min Seo Kim, Ali-Asghar Kolahi, Kewal Krishan, Narinder Kumar, Thao Thi Thu Le, Stephen S Lim, Stany W Lobo, Azeem Majeed, Ahmad Azam Malik, Mohamed Kamal Mesregah, Tomislav Mestrovic, Erkin M Mirrakhimov, Manish Mishra, Arup Kumar Misra, Madeline E Moberg, Nouh Saad Mohamed, Syam Mohan, Ali H Mokdad, Kaveh Momenzadeh, Mohammad Ali Moni, Yousef Moradi, Vincent Mougin, Satinath Mukhopadhyay, Christopher J L Murray, Sreenivas Narasimha Swamy, Van Thanh Nguyen, Robina Khan Niazi, Mayowa O Owolabi, Jagadish Rao Padubidri, Jay Patel, Shrikant Pawar, Paolo Pedersini, Quinn Rafferty, Mosiur Rahman, Mohammad-Mahdi Rashidi, Salman Rawaf, Aly M A Saad, Amirhossein Sahebkar, Fatemeh Saheb Sharif-Askari, Mohamed Metwalii Khalifa Saleh, Austin E Schumacher, Allen Seylani, Paramdeep Singh, Amanda E Smith, Ranjan Solanki, Yonatan Solomon, Ker-Kan Tan, Nathan Y Tat, Nigusie Selomon Selomon Tibebu, Yuyi You, Peng Zheng, Osama A Zitoun, Theo Vos, Lyn M March, Anthony D Woolf

## Abstract

**Background:**

Gout is an inflammatory arthritis manifesting as acute episodes of severe joint pain and swelling, which can progress to chronic tophaceous or chronic erosive gout, or both. Here, we present the most up-to-date global, regional, and national estimates for prevalence and years lived with disability (YLDs) due to gout by sex, age, and location from the Global Burden of Diseases, Injuries, and Risk Factors Study (GBD) 2021, as well as forecasted prevalence to 2050.

**Methods:**

Gout prevalence and YLDs from 1990 to 2020 were estimated by drawing on population-based data from 35 countries and claims data from the USA and Taiwan (province of China). Nested Bayesian meta-regression models were used to estimate prevalence and YLDs due to gout by age, sex, and location. Prevalence was forecast to 2050 with a mixed-effects model.

**Findings:**

In 2020, 55·8 million (95% uncertainty interval 44·4–69·8) people globally had gout, with an age-standardised prevalence of 659·3 (525·4–822·3) per 100 000, an increase of 22·5% (20·9–24·2) since 1990. Globally, the prevalence of gout in 2020 was 3·26 (3·11–3·39) times higher in males than in females and increased with age. The total number of prevalent cases of gout is estimated to reach 95·8 million (81·1–116) in 2050, with population growth being the largest contributor to this increase and only a very small contribution from the forecasted change in gout prevalence. Age-standardised gout prevalence in 2050 is forecast to be 667 (531–830) per 100 000 population. The global age-standardised YLD rate of gout was 20·5 (14·4–28·2) per 100 000 population in 2020. High BMI accounted for 34·3% (27·7–40·6) of YLDs due to gout and kidney dysfunction accounted for 11·8% (9·3–14·2).

**Interpretation:**

Our forecasting model estimates that the number of individuals with gout will increase by more than 70% from 2020 to 2050, primarily due to population growth and ageing. With the association between gout disability and high BMI, dietary and lifestyle modifications focusing on bodyweight reduction are needed at the population level to reduce the burden of gout along with access to interventions to prevent and control flares. Despite the rigour of the standardised GBD methodology and modelling, in many countries, particularly low-income and middle-income countries, estimates are based on modelled rather than primary data and are also lacking severity and disability estimates. We strongly encourage the collection of these data to be included in future GBD iterations.

**Funding:**

Bill & Melinda Gates Foundation and the Global Alliance for Musculoskeletal Health.

## Introduction

Gout is the most common form of inflammatory arthritis,^1^ manifesting as acute flares of severe joint pain, swelling, redness, and warmth in one or more joints, which can progress to chronic destructive arthropathy. The prevalence of gout is higher in males than females, and increases with age.^2^ Although a high serum urate concentration is the most important risk factor for the development of gout,^2^ genetic factors have a strong influence on the occurrence of gout, and a range of risk factors, such as medications, comorbidities, and environmental exposures, are also implicated.^3^, ^4^ Many factors that contribute to hyperuricaemia are also risk factors for incident gout, including obesity, metabolic syndrome, and chronic kidney disease, factors commonly seen in younger people with gout. Gout has been associated with cardiovascular, metabolic, and renal comorbidities.^5^

Effective management strategies for gout include treatment of the acute flares and ameliorating the long-term consequences that contribute to disability.^6^ Current recommendations for managing acute flares include the use of non-steroidal anti-inflammatory drugs (NSAIDs), colchicine, or glucocorticoids. To prevent recurrent gout flares, the primary goal is to reduce serum urate concentrations, and lifelong administration of urate-lowering drugs is crucial. Lifestyle modifications (eg, bodyweight loss) and educational programmes focusing on dietary improvement could also be useful for long-term prevention of episodes or flares in people with gout.^2^

Gout can lead to reduced mobility and impaired physical function, contributing to work absenteeism.^7^ In addition to its physical impacts, gout imposes an economic cost on those with the condition, with costs rising with increasing serum urate concentrations and the number of flares.^8^, ^9^ Untreated gout places a substantial burden on the global health system, as chronic gout causes tophi formation, chronic joint pain, and erosion and damage to joints, resulting in an increase in morbidity and disability-adjusted life-years (DALYs). Furthermore, despite advances in the treatment of gout, it is still underdiagnosed and undertreated,^10^ and is associated with an increased risk of mortality and comorbidities.^11^, ^12^ Therefore, quantifying the burden and pattern of gout cases by age and sex, as well as making projections for the future, are necessary to efficiently target the current and future needs of the population. We aimed to report the global, regional, and national burden and trends associated with gout and the burden attributed to risk factors, in terms of prevalence and age-standardised rates, from 1990 to 2020, as well as projections up to 2050.

**Research in context**

**Evidence before this study**

The Global Burden of Diseases, Injuries, and Risk Factors Study (GBD) has reported on the incidence, prevalence, and mortality of 371 diseases. GBD comprehensively provides estimates of levels and trends in prevalence and years lived with disability (YLDs) for gout and its attributable risk factors, by age and sex. Recent publications on GBD data for gout are available; however, one used GBD 2017 data and has been superseded by two further rounds of GBD, and the other was outside the GBD collaboration and did not have access to latest GBD modelling. Additionally, these publications did not present projections incorporating the demographic and epidemiological drivers of the global burden of gout.

**Added value of this study**

This study provides an update on the burden of gout to 2020 and provides projections of the prevalence of gout at the global and regional level up to 2050. Using a model that includes updated covariates, we estimated that, in 2020, there were 55·8 million (95% uncertainty interval 44·4–69·8) people with gout, and the age-standardised prevalence rate has increased by 22·5% (20·9–24·2) globally since 1990. The number of individuals with gout is projected to reach 95·8 million (81·1–116) by 2050, with approximately 0·6% of the forecasted increase in case numbers due to changes in the prevalence of gout. The burden of gout was higher within high-income regions. High BMI (>20–25 kg/m^2^) accounted for 34·3% (27·7–40·6) of age-standardised gout YLDs and kidney dysfunction accounted for 11·8% (9·3–14·2).

**Implications of all the available evidence**

Gout continues to cause a considerable burden in all regions, with the burden increasing with advancing age despite modifiable risk factors and effective interventions to control hyperuricaemia and treat gout flares. We estimated that the total prevalent cases of gout will increase by more than 70% from 2020 to 2050. Apart from population growth, ageing and an increasing rate of disease will contribute the largest number of prevalent cases in most regions up to 2050. To reduce the burden of gout, risk factors such as high BMI should be addressed, with timely access and adherence to treatment to prevent the transition from acute to chronic tophaceous or chronic erosive gout, or both. Reducing other risk factors such as alcohol and sugar-sweetened beverage intake and consumption of purine-rich food, not currently quantified in GBD modelling for gout, might also reduce the burden of gout.

## Methods

### Overview

This Article was produced as part of the Global Burden of Diseases, Injuries, and Risk Factors Study (GBD) Collaborator Network and in accordance with the GBD Protocol. The Guidelines for Accurate and Transparent Health Estimates Reporting (GATHER) statement were followed. GBD is the largest and the most comprehensive effort to estimate the burden of disease across the world. GBD 2021, the most recent iteration of the project, assessed the burden of 371 diseases and injuries and 88 risk factors for 204 countries and territories during the period 1990–2021. Detailed descriptions of the methodology for estimating the burden of diseases and risk factors can be found elsewhere.^13^, ^14^, ^15^ Data are reported by super-region, region, and country, with super-regions based on epidemiological similarity and geographical closeness. Where data for individual locations were not available, prevalence estimates were modelled with available data from super-region priors. DisMod-MR 2.1, a Bayesian meta-regression tool, is run as a geographical cascade. First, a model is run with all global data. Using random effects and predictive covariates, the results of this initial model are passed down to the next level models by super-region as Bayesian priors. This is repeated for regional, country, and, where applicable, subnational models.

### Case definition and input data

Physician-diagnosed gout based on the criteria of the American College of Rheumatology (ACR) was used as the reference case definition in GBD.^14^, ^16^ Data sources that used diagnostic criteria other than the reference criteria (such as self-reported gout or gout identified through administrative data) were adjusted with a meta-regression tool, MR-BRT (Meta-Regression Bayesian Regularised Trimmed), as described elsewhere.^14^

The most recent systematic review on gout was conducted for GBD 2013. MEDLINE, EMBASE, CINAHL, CAB Abstracts, and the WHO Library (WHOLIS) were searched from 1980 to 2009 with relevant search terms and appropriate combinations (appendix p 1), which have been detailed in a previous publication.^14^ Studies with non-representative samples, small sample sizes, reviews, and studies that did not use a population-based approach were not included. Since the last systematic review, 15 additional studies (which were shared through the GBD Collaborator Network or prospectively found through a targeted search), claims data from the USA for 2000 and 2010–14 (by state), and claims data from Taiwan (province of China) for 2016 were added. GBD 2021 used the M10 code from version 10 of the International Classification of Diseases (ICD) and the corresponding ICD-9-CM diagnosis code (274: Gout) to identify cases of gout in medical claims data.

There were 35 countries with data on gout, and 130 source-years were used to estimate the burden of gout. Detailed information about the data sources used to estimate the prevalence of gout are provided in the appendix (pp 4–5).^14^

### Data processing and disease modelling

Input data were extracted by age and sex where available. Where the prevalence rates were reported for broad age groups by sex and separately by specific age groups for male and female sexes combined, age-specific estimates were split by sex with the reported sex ratio and bounds of uncertainty. In cases where the combined prevalence data could not be split with a within-study ratio, a sex ratio was derived from a meta-analysis of existing sex-specific data, using the MR-BRT tool.^14^ Regression results were used to split the combined male and female sex data into sex-specific rates. The female-to-male ratio was 0·33 (95% uncertainty interval [UI] 0·33–0·34), denoting higher prevalence in males. Where studies reported estimates across age groups spanning 25 years or more, these were split into 5-year age groups using the global prevalence age pattern estimated in the previous iteration of the study, GBD 2019. Additionally, MR-BRT was used to adjust for alternative case definitions, including self-reported gout and gout identified through administrative data and claims data in the USA from the years 2000 and 2010–16 and Taiwan (province of China) in 2016.^14^

The non-fatal burden of gout was modelled with DisMod-MR 2.1, a Bayesian meta-regression tool.^14^ The modelling process assumed that there was no excess mortality or remission (defined as complete cure) associated with gout. Prevalence and annual incidence estimates were generated by age, sex, location, and year. The model included one country-level covariate, the gout summary exposure value (SEV) scalar, a normalised value of risks for a disease. In the case of gout, these risks are kidney dysfunction and high BMI. High BMI is defined as greater than 20–25 kg/m^2^, whereas kidney dysfunction is defined as an estimated glomerular filtration rate (eGFR) less than 60 mL/min per 1·73 m^2^ or an albumin to creatinine ratio greater than or equal to 30 mg/g.

Gout severity was classified as asymptomatic, acute gout episode, and polyarticular tophaceous gout, with corresponding disability weights shown in the appendix (p 3). Asymptomatic gout includes those who have a diagnosis of gout but do not have pain or functional difficulties. These severity categories represent the natural history of gout.

The number and duration of gout flares were estimated from previously published studies (appendix p 2). Data from three studies on the distribution of the number of gout flares per year were used to fit a lognormal curve with a least squared differences method, with the estimated average number of gout flares being 5·66 per year (95% UI 5·14–6·18).^17^, ^18^, ^19^, ^20^, ^21^ The results of two studies were averaged to find the average duration of flares, which was 6·1 days (5·4–6·8).^18^, ^19^ These two estimates were multiplied and then divided by the number of days in a year to produce the proportion of symptomatic time for acute gout, which was 9·4% (8·0–10·9). As there were no data on those with chronic tophaceous or chronic erosive gout, or a combination of both, we assumed the proportion to be the same as those who had at least 52 flares a year (ie, at least once a week) as implied by the lognormal curve. The prevalence estimate of each sequela was multiplied with its sequela-specific disability weight to produce the years lived with disability (YLDs).

Age-standardised rates were computed with the GBD standard population. All mean and uncertainty estimates were produced by taking the final 100 outputs from the posterior distribution after model convergence (termed draws), collapsing to the mean and 95% UIs as the 2·5th and 97·5th percentiles of the draws. Count data are presented to three significant figures, while rates and percentages are presented to one decimal place.

### Risk estimation

Two potential risk factors for developing gout, high BMI and impaired kidney function, had sufficient epidemiological evidence from population-based data to be included in the modelling process to assess attributable risk for developing gout.^13^ For high BMI, exposure is defined as BMI greater than a theoretical minimum risk exposure level, which GBD defines as 20–25 kg/m^2^ for adults aged 20 years and older, and the exposure was calculated with spatiotemporal Gaussian process regression, as well as mixed-effects models. For impaired kidney function, risk exposure is the presence of kidney dysfunction with a theoretical minimum risk exposure level of zero (ie, no kidney dysfunction), and thus exposure was obtained from the prevalence of those with eGFR less than 60 mL/min per 1·73 m^2^. The relative risks posed as these exposures worsen (through stages 3–5 of chronic kidney disease) were ascertained from a meta-analysis of the epidemiological evidence with the meta-regression tool MR-BRT. The population attributable fraction was calculated as the proportional reduction in gout that would result if exposure to these risk factors was reduced to the theoretical minimum risk exposure level. Following this, the number of YLDs due to gout attributable to each risk factor was derived by multiplying the total YLDs for gout by the corresponding population attributable fractions for each age group, sex, location, and year. The definition of these risk factors and information on estimating the population attributable fractions and their attributable burden for each risk factor have been described in detail previously.^13^

### Estimate projections

The number of global and regional cases of gout were estimated to the year 2050 with forecasted population estimates^22^ and a regression to forecast prevalence with the Socio-demographic Index (SDI) as a predictor, since health outcomes are closely tied to SDI. Forecasting models do not take into account specific risk factors or change in case identification over time. Age-specific, location-specific, and sex-specific GBD 2019 prevalence rates for estimation years from 1990 to 2020 were logit transformed and used in the following regression model:

$$E[\text{logit}(Y_{l,a,s,\gamma })]=\beta_{1}SDI+\alpha_{l,a,s}$$

In this linear model fitted to logit-transformed prevalence, the term on the left side of the equation is the forecasted logit(prevalence) estimated by (*l,a,s,y*), the unique location-age-sex-year in which β_1_ is the fixed coefficient on SDI over time, and α_l,a,s_ is the location-age-sex-specific random intercept. To compute forecasted cases, forecasted rates were multiplied by forecasted population values.^22^ Forecasted prevalence rates were intercept-shifted to GBD prevalence in the year 2021 and this difference was used to shift all forecasted values to the year 2050. Validation testing was conducted with osteoarthritis estimates from 1990 to 2010 to project prevalence from 2010 to 2019 by age, sex, location, and year (appendix p 4). A Das Gupta decomposition analysis was performed to ascertain the relative contributions to the change in case number, between 2020 and 2050, of population growth, population ageing, and changes in prevalence unrelated to demographics.^23^

### Role of the funding source

The funders of the study had no role in study design, data collection, data analysis, data interpretation, writing of the report, or the decision to submit the manuscript for publication.

## Results

**Figure 1:**
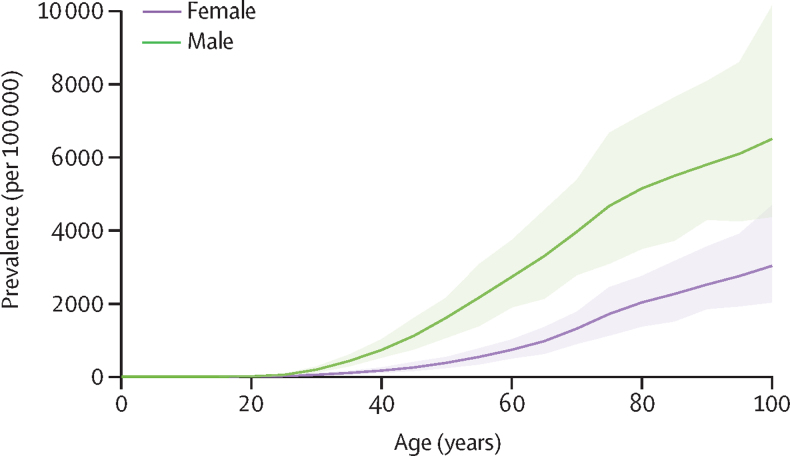
Global prevalence of gout by age and sex in 2020 Shaded areas represent 95% uncertainty intervals.

A total of 131 sources were used in the current analysis, spanning 35 countries over 15 regions (appendix pp 4–5). In 2020, there were an estimated 55·8 million (95% UI 44·4–69·8) people with gout globally, a substantial increase (150·6% [142·7–159·2]) from the 1990 estimates. The global age-standardised prevalence of gout in 2020 was 659·3 (525·4–822·3) per 100 000. This was an increase of 22·5% (20·9–24·2) between 1990 and 2020 (table). The global prevalence of gout was found to be 3·26 (3·11–3·39) times higher in males than females, with a global age-standardised prevalence rate of 1030·8 (823·4–1289·5) per 100 000 for males and 316·4 (249·1–394·3) per 100 000 for females in 2020. Prevalence also increased with age (figure 1).

**Table:** Prevalence and age-standardised rate of years lived with disability due to gout in 2020, and percentage change from 1990 globally and for GBD regions

	**Number of prevalent cases**	**Percentage change in number of prevalent cases from 1990 to 2020**	**Age-standardised prevalence rate per 100 000**	**Percentage change in age-standardised prevalence rate from 1990 to 2020**	**Number of YLDs**	**Age-standardised rate of YLDs per 100 000 in 2020**	**Percentage change in age-standardised rate of YLDs per 100 000 from 1990 to 2020**
**Global**	**55 800 000 (44 400 000 to 69 800 000)**	**150·6% (142·7 to 159·2)**	**659·3 (525·4 to 822·3)**	**22·5% (20·9 to 24·2)**	**1 730 000 (1 220 000 to 2 390 000)**	**20·5 (14·4 to 28·2)**	**22·0% (19·8 to 24·2)**
Male	41 700 000 (33 100 000 to 52 800 000)	150·5% (142·1 to 160·2)	1030·8 (823·4 to 1289·5)	21·6% (20·0 to 23·4)	1 300 000 (909 000 to 1 800 000)	32·1 (22·6 to 44·1)	21·4% (19·3 to 23·6)
Female	14 100 000 (11 000 000 to 17 500 000)	151·0% (143·7 to 157·8)	316·4 (249·1 to 394·3)	20·8% (19·0 to 22·8)	429 000 (304 000 to 587 000)	9·7 (6·8 to 13·2)	20·1% (17·5 to 22·5)
**Central Europe, eastern Europe, and central Asia**	**2 490 000 (1 940 000 to 3 150 000)**	**49·1% (45·1 to 52·8)**	**411·4 (319·8 to 518·3)**	**15·0% (13·6 to 16·3)**	**76 500 (53 400 to 105 000)**	**12·7 (8·8 to 17·4)**	**15·1% (12·0 to 18·0)**
Central Asia	365 000 (283 000 to 468 000)	97·3% (89·7 to 103·9)	441·1 (340·4 to 550·0)	16·5% (13·1 to 19·3)	11 600 (7 890 to 15 900)	13·8 (9·5 to 18·8)	16·5% (10·5 to 22·5)
Central Europe	709 000 (551 000 to 911 000)	54·5% (48·6 to 60·7)	364·4 (287·1 to 461·8)	14·5% (12·2 to 16·2)	21 700 (15 100 to 30 000)	11·3 (7·9 to 15·4)	14·7% (9·5 to 18·8)
Eastern Europe	1 410 000 (1 110 000 to 1 780 000)	37·9% (33·9 to 42·2)	430·3 (334·5 to 541·2)	14·8% (13·1 to 16·5)	43 200 (30 300 to 59 600)	13·3 (9·2 to 18·2)	14·7% (10·9 to 18·1)
**High income**	**18 700 000 (15 300 000 to 23 000 000)**	**132·3% (124·0 to 145·5)**	**1025·9 (845·9 to 1272·0)**	**44·3% (39·1 to 50·9)**	**570 000 (407 000 to 771 000)**	**31·7 (22·8 to 42·9)**	**43·2% (38·4 to 50·2)**
Australasia	669 000 (520 000 to 873 000)	168·8% (150·2 to 194·1)	1424·4 (1129·6 to 1853·8)	32·3% (23·5 to 44·3)	20 400 (14 600 to 28 600)	43·9 (30·9 to 60·8)	31·8% (21·1 to 42·5)
High-income Asia Pacific	2 700 000 (2 080 000 to 3 440 000)	105·6% (92·9 to 121·1)	728·4 (576·1 to 933·3)	13·1% (11·0 to 15·5)	83 500 (58 400 to 116 000)	22·9 (16·0 to 31·6)	13·3% (9·6 to 17·4)
High-income North America	9 680 000 (8 130 000 to 11 600 000)	199·6% (181·7 to 225·7)	1719·8 (1450·2 to 2078·7)	76·6% (65·0 to 90·7)	291 000 (210 000 to 394 000)	52·5 (37·2 to 70·6)	74·0% (61·7 to 87·3)
Southern Latin America	759 000 (601 000 to 974 000)	118·1% (106·6 to 128·9)	926·0 (739·8 to 1196·5)	23·4% (17·9 to 29·2)	23 700 (16 100 to 33 100)	29·0 (19·9 to 40·6)	23·1% (13·7 to 31·8)
Western Europe	4 920 000 (3 770 000 to 6 240 000)	68·5% (63·5 to 73·9)	627·3 (499·5 to 802·7)	14·6% (11·8 to 16·4)	151 000 (105 000 to 207 000)	19·6 (13·5 to 26·7)	14·7% (11·1 to 18·9)
**Latin America and Caribbean**	**1 430 000 (1 130 000 to 1 780 000)**	**204·1% (190·4 to 218·6)**	**230·6 (183·2 to 285·2)**	**24·4% (23·2 to 26·1)**	**45 100 (31 100 to 61 500)**	**7·2 (5·0 to 9·9)**	**23·6% (19·5 to 26·8)**
Andean Latin America	175 000 (138 000 to 221 000)	238·8% (224·5 to 256·0)	289·6 (229·6 to 365·5)	30·4% (25·1 to 37·4)	5530 (3590 to 7580)	9·1 (6·0 to 12·5)	28·6% (15·5 to 42·1)
Caribbean	129 000 (104 000 to 157 000)	137·6% (126·8 to 148·0)	245·7 (198·1 to 300·8)	24·4% (21·0 to 27·8)	4060 (2850 to 5560)	7·8 (5·4 to 10·6)	23·4% (14·5 to 34·9)
Central Latin America	478 000 (378 000 to 589 000)	205·8% (189·1 to 223·3)	188·4 (148·6 to 231·1)	21·4% (19·7 to 23·3)	15 300 (10 400 to 21 200)	6·0 (4·1 to 8·3)	21·1% (15·4 to 27·2)
Tropical Latin America	648 000 (513 000 to 812 000)	211·4% (198·2 to 228·4)	254·7 (203·0 to 316·5)	26·4% (24·1 to 28·9)	20 200 (14 200 to 27 400)	7·9 (5·6 to 10·7)	25·5% (18·2 to 32·0)
**North Africa and Middle East**	**2 610 000 (2 050 000 to 3 310 000)**	**218·1% (210·5 to 225·8)**	**525·9 (411·5 to 657·3)**	**20·1% (18·1 to 22·2)**	**81 800 (56 100 to 114 000)**	**16·2 (11·4 to 22·2)**	**19·2% (14·6 to 23·0)**
**South Asia**	**6 410 000 (5 000 000 to 8 170 000)**	**163·9% (154·5 to 173·5)**	**421·2 (328·6 to 528·9)**	**7·8% (6·4 to 9·4)**	**199 000 (138 000 to 275 000)**	**12·9 (9·1 to 17·9)**	**8·8% (5·4 to 12·0)**
**Southeast Asia, east Asia, and Oceania**	**21 700 000 (16 800 000 to 27 600 000)**	**177·5% (161·0 to 193·3)**	**774·2 (606·4 to 972·2)**	**25·1% (22·4 to 27·3)**	**682 000 (466 000 to 947 000)**	**24·3 (16·8 to 33·5)**	**24·8% (21·0 to 27·7)**
East Asia	17 300 000 (13 300 000 to 21 900 000)	175·9% (157·2 to 194·1)	817·4 (641·2 to 1023·9)	26·4% (23·4 to 29·1)	542 000 (372 000 to 754 000)	25·7 (17·8 to 35·5)	26·0% (21·6 to 29·4)
Oceania	59 500 (46 700 to 76 500)	166·6% (154·7 to 178·1)	701·5 (555·1 to 888·3)	8·0% (3·9 to 12·4)	1870 (1290 to 2730)	21·6 (15·4 to 30·3)	7·5% (−1·6 to 16·9)
Southeast Asia	4 350 000 (3 390 000 to 5 600 000)	184·5% (172·5 to 192·9)	642·1 (501·3 to 816·6)	21·3% (18·9 to 23·4)	138 000 (92 700 to 190 000)	20·2 (13·8 to 27·8)	22·0% (17·7 to 26·3)
**Sub-Saharan Africa**	**2 470 000 (1 940 000 to 3 140 000)**	**144·9% (140·7 to 149·4)**	**455·9 (354·4 to 568·3)**	**4·8% (3·8 to 5·9)**	**77 700 (53 700 to 107 000)**	**14·0 (9·9 to 19·5)**	**5·1% (2·9 to 7·3)**
Central sub-Saharan Africa	274 000 (216 000 to 346 000)	163·0% (146·8 to 175·2)	429·9 (334·3 to 535·5)	2·0% (−3·6 to 7·1)	8540 (5950 to 12 100)	13·1 (9·2 to 18·4)	2·6% (−6·6 to 12·2)
Eastern sub-Saharan Africa	866 000 (682 000 to 1 100 000)	149·6% (143·1 to 155·5)	450·3 (351·4 to 563·4)	6·0% (3·7 to 7·4)	27 300 (19 000 to 37 700)	13·9 (9·9 to 19·2)	6·6% (2·7 to 9·9)
Southern sub-Saharan Africa	342 000 (265 000 to 436 000)	129·7% (124·8 to 134·5)	549·5 (425·1 to 684·6)	9·1% (6·9 to 11·0)	10 600 (7250 to 14 700)	16·8 (11·7 to 23·4)	8·0% (3·0 to 12·7)
Western sub-Saharan Africa	986 000 (775 000 to 1 250 000)	141·8% (136·3 to 147·4)	440·7 (341·9 to 550·4)	3·5% (1·7 to 5·0)	31 200 (21 300 to 43 500)	13·6 (9·5 to 19·1)	3·9% (1·1 to 6·4)

Data in parentheses are 95% uncertainty intervals. Count data are presented to three significant figures, while rates and percentages are presented to one decimal place. Region and super-region numbers do not sum to the global prevalence due to rounding and modelling adjustments for countries with populations below 50 000. GBD=Global Burden of Diseases, Injuries, and Risk Factors Study. YLDs=years lived with disability.

**Figure 2:**
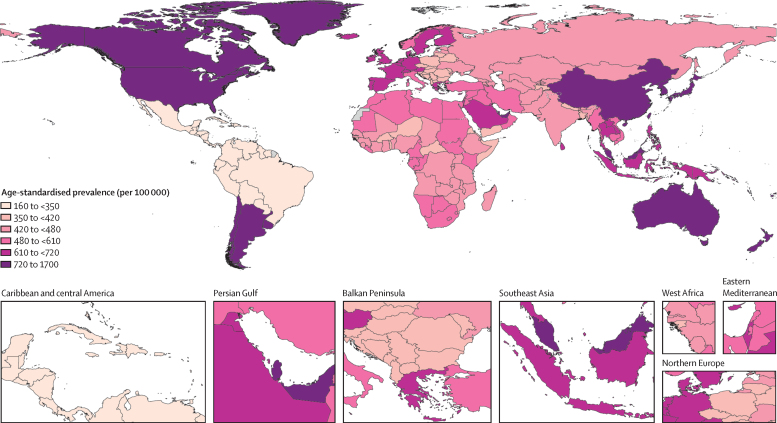
Age-standardised prevalence of gout by country for male and female sexes combined and all ages in 2020

In 2020, the age-standardised prevalence of gout was highest in the regions of high-income North America (1719·8 [95% UI 1450·2–2078·7] per 100 000), Australasia (1424·4 [1129·6–1853·8] per 100 000), and southern Latin America (926·0 [739·8–1196·5] per 100 000). By contrast, Tropical Latin America (254·7 [203·0–316·5] per 100 000), the Caribbean (245·7 [198·1–300·8] per 100 000), and central Latin America (188·4 [148·6–231·1] per 100 000) showed the lowest age-standardised prevalence (table). Country-level age-standardised prevalence estimates are shown in figure 2 and the appendix (pp 6–18).

From 1990 to 2020, age-standardised prevalence increased substantially in most regions, with the largest increases seen in high-income North America (76·6% [95% UI 65·0–90·7]), Australasia (32·3% [23·5–44·3]), and Andean Latin America (30·4% [25·1–37·4]; table).

Globally, gout accounted for 1·73 million (95% UI 1·22–2·39) YLDs in 2020, with an age-standardised rate of 20·5 (14·4–28·2) per 100 000, which was 22·0% (19·8–24·2) higher than in 1990 (table). The largest increases in the age-standardised YLD rate from 1990 to 2020 were seen in high-income North America (74·0% [61·7–87·3]), Australasia (31·8% [21·1–42·5]), and Andean Latin America (28·6% [15·5–42·1]; table). A positive association was observed between the age-standardised YLD rate of gout and level of development, with high-income regions showing higher YLD rates and a greater change since 1990 than low-income and middle-income regions (table).

Globally, in 2020, high BMI accounted for 34·3% (95% UI 27·7–40·6) of the YLDs due to gout and kidney dysfunction accounted for 11·8% (9·3–14·2). YLDs attributable to risk factors were higher in females than in males for high BMI (36·2% [29·5–42·9] *vs* 33·6% [27·0–39·8]) and for kidney dysfunction (14·6% [11·5–17·2] *vs* 11·3% [8·9–13·6]). The high BMI-attributable burden of gout ranged from 20·2% (16·7–24·3) in south Asia to 46·6% (37·7–54·9) in high-income North America. The attributable gout burden for kidney dysfunction ranged from 7·0% (5·4–8·6) in eastern sub-Saharan Africa to 18·8% (15·1–22·4) in central sub-Saharan Africa (appendix p 19).

**Figure 3:**
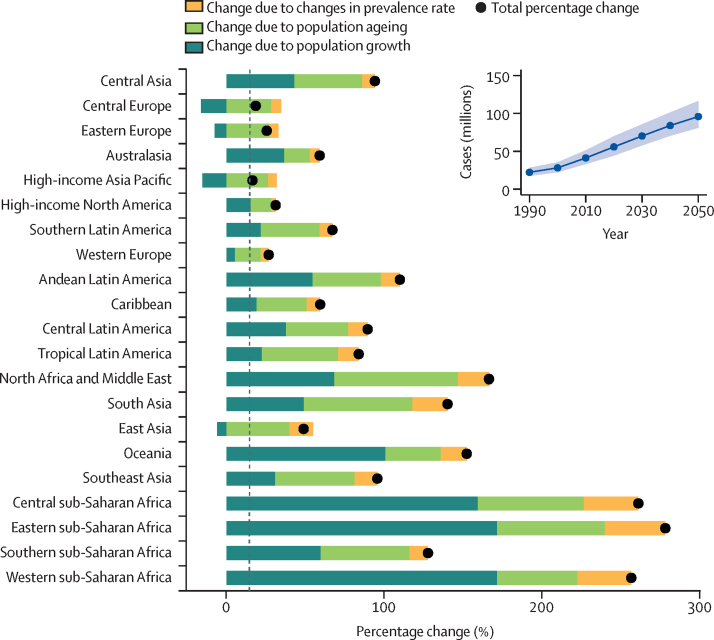
Global cases of gout forecast to year 2050 and decomposition of changes in case counts by region, 2020–50 In the inset graph, shaded areas represent 95% uncertainty intervals.

In 2050, there will be an estimated 95·8 million (95% UI 81·1–116) prevalent cases of gout globally, an increase of 72·6% (54·9–100·3) from 2020 to 2050 (figure 3; appendix p 20). Age-standardised gout prevalence in 2050 is forecast to be 667 (531–830) per 100 000 population. In 2050, high-income North America (1780 [1520–2150] per 100 000) is estimated to have the highest age-standardised prevalence of gout and central Latin America (206 [169–248] per 100 000) will have the lowest age-standardised prevalence (appendix p 20).

A decomposition analysis by region shows the relative contribution of population growth, population ageing, and changes in prevalence to the forecast increase in cases across regions (figure 3). Overall, approximately 0·6% of the forecasted increase in case numbers was due to changes in the prevalence of gout. Regionally, the increase in case numbers due to the increase in the prevalence of gout ranged from 2·7% in high-income North America to 38·2% in eastern sub-Saharan Africa.

The greatest contribution to forecast cases of gout are population ageing and population growth. Population growth was the largest contributor to gout prevalence in Oceania and the regions of sub-Saharan Africa, while population ageing was a major contributor in east Asia and the European regions.

## Discussion

Globally, the total number of people with gout doubled from 1990 to 2020, and age-standardised prevalence increased by 22·5% from 1990, with increases in most GBD regions. In line with the literature,^24^, ^25^ the burden of gout in 2020 was three times higher in males than in females in all age groups. Higher rates of gout were seen in the older age groups in this study, which might be due to the cumulative effects of monosodium urate accumulation over time, higher prevalence of kidney dysfunction, and usage of medications contributing to hyperuricaemia.^26^ Our forecasts showed that the prevalence of gout is projected to be up to 96 million in 2050, with the increase being primarily due to population growth and ageing, and only a small increase in gout prevalence.

Gout was more common in males than females. An analysis of individuals enrolled in a gout registry has shown differences in the factors that contribute to gout between males and females.^26^ Women more often have renal disease and concomitant use of thiazides or other diuretics.^26^ Men are more likely to report intake of foods associated with both the development of gout and gout flares^2^, ^25^, ^26^ and are more likely than women to have hyperuricaemia, a precursor for the development of gout.^2^

In most regions, estimates of the burden of gout increased from 1990 to 2020. Variations between the different regions can be partially explained in terms of the effects of genetic and racial factors on the incidence of gout, especially among some Indigenous populations, and potential differences in exposure to other risk factors, such as high BMI, kidney dysfunction, and consumption of red and processed meat or seafood.^2^, ^26^, ^27^ For example, Maori and Pacific Islanders in New Zealand have severe gout disproportionately compared with New Zealand Europeans, developing severe disease at a younger age, with more frequent flares, higher rates of hospital admission for gout, and more tophaceous disease, in addition to experiencing treatment disparities.^27^

Higher exposure to gout risk factors, such as kidney dysfunction and high BMI, might be partially responsible for the increases in the age-standardised YLD rate of gout in high-income regions. High-income Asia Pacific regions had the highest YLDs attributable to kidney dysfunction, where a higher prevalence of kidney dysfunction has been reported.^28^ Moreover, as the kidney dysfunction YLDs worsen with age, the burden attributable to gout also increases.^28^ The highest YLDs attributable to high BMI were seen in high-income North America.^2^, ^9^ Obesity (ie, a BMI >30 kg/m^2^) has previously been associated with a greater than two-fold increased risk of developing gout compared with a BMI less than 30 kg/m^2^.^29^ In addition to obesity, diet has been linked with an increased risk of gout, especially among those with a genetic predisposition to gout. Consumption of red meat, seafood, shellfish, fructose-sweetened drinks, and alcoholic drinks (particularly beer) increase the risk of incident gout.^29^, ^30^ These factors were independent risk factors and more than doubled the risk of incident gout compared with people without these factors. Recent studies have acknowledged the important interaction between diet and susceptibility, including a UK Biobank study that suggested a healthy lifestyle might reduce the risk of genetically predisposed gout by as much as a third.^31^, ^32^ Men who consumed two or more servings of sugar-sweetened drinks per day were found to have a higher risk of gout compared with those who consumed less than one serving monthly.^33^ Conversely, low-fat dairy products, soy foods, and coffee are associated with a reduced risk of gout.^30^ The geographical distribution of the gout burden aligns with geographical diet patterns as described in the literature. For other conditions, GBD has the ability to include dietary risks such as a diet high in red meat, high in sugar-sweetened beverages, and low in seafoods containing omega-3 fatty acids, as well as alcohol use within the modelling process, so the inclusion of these for gout modelling in future iterations of the GBD is recommended.

Although non-steroidal anti-inflammatory medications are beneficial in the treatment of acute gout flares, ongoing prevention of recurring gout can be achieved by lowering uric acid through the use of urate lowering therapy, in addition to low-dose anti-inflammatory medications.^2^ There is evidence to support a treat-to-target approach for lowering serum urate, which can suppress gout flares and prevent joint and other end organ damage in the long term by reducing the total body load of urate tissue deposits.^2^ However, it has been reported that suboptimal treatment of gout occurs in many countries, with only a third to half of patients with gout receiving urate-lowering therapy, and fewer than half of patients adhering to the long-term treatment that is needed.^34^, ^35^ Suboptimal treatment might lead to unnecessary development of disabling erosive gout. One of the most important strategies to reduce the ongoing burden of gout is improving education around adherence to treatment.^36^ The ability to do this is likely to vary across regions and might contribute to the differences seen across regions.

Our forecasts showed that the prevalence of gout is estimated to be up to 96 million in 2050. Unfortunately, these forecasts cannot yet be based on forecasted rates of risk factors including high BMI and kidney dysfunction, and SDI has been taken as a proxy in the forecasting process. It is known that global rates of overweight and obesity are increasing,^37^ so the actual forecasted prevalence rate could be higher if the increase in obesity is greater than the increase in SDI. The impact of COVID-19 on forecast estimates for gout is also not yet known. As with many other conditions, maintaining a healthy bodyweight could reduce the prevalence of gout, in addition to controlling the underlying conditions for the development of kidney impairment, such as hypertension and diabetes. Designing programmes for the early detection and treatment of kidney diseases can prevent further complications.^28^

There are some limitations that should be taken into consideration when interpreting these estimates. Although the GBD modelling process is robust, the sparsity of data on the prevalence of gout, particularly from low-income and middle-income countries, remains a major limitation. Of the 204 countries included in GBD 2021, data were available from just 35 countries covering 15 regions, and a large proportion of the modelling input was from claims data from the USA and Taiwan (province of China). There are wide uncertainty intervals in the estimated prevalence in almost all regions, indicating lower certainty in the estimates presented. We emphasise the need for more thorough data collection, in particular in low-income and middle-income regions, so that the estimates derived from the modelling process can be based on more primary data sources.

Although GBD modelling adjusts for varying case definitions, diagnosis and management of gout relies on access to suitable laboratory facilities for the measurement of serum urate concentrations, which might not be readily available in all countries. The clinical presentation of gout overlaps with acute episodes of calcium pyrophosphate crystal arthritis (known as pseudogout), which could lead to overestimates. Although the symptoms are similar, gout is caused by monosodium urate crystals while calcium pyrophosphate crystal arthritis is caused by calcium pyrophosphate crystals. The distinction between these two conditions in the clinical setting is often challenging and requires further radiological and pathological assessment of joint and synovial fluid, which is not always possible in low-income settings. Nevertheless, both conditions lead to pain and disability that could become chronic if untreated. The current study utilised physician diagnosis based on the 1977 American Rheumatism Association (now ACR) criteria as the reference case definition of gout; however, as data based on more recent criteria such as the 2015 ACR–European Alliance of Associations for Rheumatology criteria for gout become available, which incorporate newer imaging modalities and improved performance characteristics, these could be used in future GBD iterations. Attributing burden to conditions that have intermittent episodes is somewhat challenging. Unless a deformity exists that would lead to some loss of function or pain, GBD methodology does not attribute disability. If there are no symptoms between flare episodes, then there is assumed to be no disability, hence the resulting estimates on the burden of gout might be an underestimate of the true burden.

The GBD model does not provide differentiation based on access to treatment, adherence, or the capacity to treat comorbid conditions without the use of drugs that can trigger gout flares. With few studies available, severity levels and disability weights used to calculate YLDs were applied consistently across regions with no regional difference being taken into consideration. Additionally, there were sparse data on the number and duration of gout flares across regions, so the GBD model has assumed all regions are the same, despite access to treatment varying across regions.

Another limitation is that no mortality was attributed to gout in the GBD model. Attributing mortality is complex in a condition that is associated with multimorbidity. GBD follows the principles of ICD to assign deaths to an underlying cause. Even if people with gout have a higher risk of death due to comorbid conditions, this does not mean deaths can be assigned to gout as the underlying cause. Having untreated gout and high concentrations of serum urate are a risk factor for all-cause mortality, and people with heart disease plus gout are perhaps more likely to die than people with heart disease alone, although gout would rarely be listed as a primary cause of death.

A further limitation is the use of high BMI and kidney dysfunction as the only risk factors. These were the only risk factors that had sufficient population-based evidence of a causal relationship with gout as required for GBD studies. Other risk factors for hyperuricaemia, which increases the risk of gout, include fasting plasma glucose, genetics, cardiovascular disease, diabetes, alcohol consumption, and dietary factors including purine-rich foods such as red meat and seafood intake in addition to the consumption of fructose-sweetened beverages.^2^ Current GBD methods for other conditions such as cardiovascular disease include a diet high in red meat and processed meat in addition to alcohol consumption as risk factors;^38^ however, the causal evidence for gout due to these risks has not yet been evaluated for inclusion in future iterations of GBD.

In conclusion, the burden of gout increased globally over the past 30 years and is forecast to continue increasing over the next three decades. It is important to note that all regions had an increase in rates of the age-standardised burden due to gout from 1990 to 2020, despite large regional variations. Our findings highlight the need to focus on the prevention and management of gout as the population ages, especially among males. Preventing the disease requires public policy interventions to control risk factors, in particular high BMI, and guide resource allocation to enable early diagnosis and access and adherence to treatment.

### GBD 2021 Gout Collaborators

### Affiliations

### Contributors

### Data sharing

The findings of this study are supported by data available in public online repositories, data publicly available upon request of the data provider, and data not publicly available due to restrictions by the data provider. Non-publicly available data were used under license for the current study but may be available from the authors upon reasonable request and with permission of the data provider. Data sources used in this analysis are listed in the appendix (pp 21–28).

## Declaration of interests

R Buchbinder reports grants or contracts from Australian National Health and Medical Research Council, Australian Government, Arthritis Australia, HCF Foundation, Cabrini Foundation; royalties from Wolters Klewer Health for authorship of the chapter*Plantar fasciitis* in *UpToDate*; all outside the submitted work. S Das reports voluntary member and leadership of the Association of Diagnostic and Laboratory Medicine and Women in Global Health, outside the submitted work. D R Kopansky-Giles reports support for the present manuscript from Global Alliance for Musculoskeletal Health; leadership or fiduciary roles in board, society, committee or advocacy groups, paid or unpaid, with Global Alliance for Musculoskeletal Health with the International Coordinating Council and World Spine Care, Canada, as a member of the Canadian Board of Directors; all outside the submitted work. K Krishan reports non-financial support from the UGC Centre of Advanced Study, CAS II (awarded to the Department of Anthropology, Panjab University, Chandigarh, India), outside the submitted work. L M March reports grants or contracts from National Health and Medical Research Council (NHMRC) Australian Government for Centre of Research Excellence for Better Outcomes in Inflammatory Arthritis, Medical Research Future Fund (MRFF) Australian Government for a Biologic Tapering trial in Adults with rheumatoid arthritis and psoriatic arthritis, and MRFF Australian Government for a biologic tapering trial in children with juvenile idiopathic arthritis; royalties from Wolters Klewer Health for authorship of the chapter *Epidemiology and risk factors for osteoarthritis* in *UpToDate*; royalties from Elsevier for being co-editor of *The Musculoskeletal System, 3rd Edn, 2022*; participation on a Data Safety Monitoring Board (DSMB) or Advisory Board with NHMRC Australian Government as a pro-bono member of DSMB for an investigator initiated text message study for low back pain; leadership or fiduciary roles in board, society, committee or advocacy groups, paid or unpaid, with Australian Rheumatology Association as a pro-bono Chair Research Advisory Committee and Global Alliance for MSK Health – Global Alliance for Musculoskeletal Health as a pro-bono Executive Committee member; all outside the submitted work. All other authors declare no competing interests.

## References

[1] Singh JA, Gaffo A (2020). Gout epidemiology and comorbidities. Semin Arthritis Rheum.

[2] Dalbeth N, Gosling AL, Gaffo A, Abhishek A (2021). Gout. Lancet.

[3] Kuo C-F, Grainge MJ, Zhang W, Doherty M (2015). Global epidemiology of gout: prevalence, incidence and risk factors. Nat Rev Rheumatol.

[4] Saag KG, Choi H (2006). Epidemiology, risk factors, and lifestyle modifications for gout. Arthritis Res Ther.

[5] Zhu Y, Pandya BJ, Choi HK (2012). Comorbidities of gout and hyperuricemia in the US general population: NHANES 2007–2008. Am J Med.

[6] Stamp LK, Farquhar H (2021). Treatment advances in gout. Best Pract Res Clin Rheumatol.

[7] Murdoch R, Barry MJ, Choi HK (2021). Gout, Hyperuricaemia and Crystal-Associated Disease Network (G-CAN) common language definition of gout. RMD Open.

[8] Rai SK, Burns LC, De Vera MA, Haji A, Giustini D, Choi HK (2015). The economic burden of gout: a systematic review. Semin Arthritis Rheum.

[9] Shields GE, Beard SM (2015). A systematic review of the economic and humanistic burden of gout. PharmacoEconomics.

[10] Rai SK, Choi HK, Choi SHJ, Townsend AF, Shojania K, De Vera MA (2018). Key barriers to gout care: a systematic review and thematic synthesis of qualitative studies. Rheumatology.

[11] Bevis M, Blagojevic-Bucknall M, Mallen C, Hider S, Roddy E (2018). Comorbidity clusters in people with gout: an observational cohort study with linked medical record review. Rheumatology.

[12] Cipolletta E, Tata LJ, Nakafero G, Avery AJ, Mamas MA, Abhishek A (2022). Association between gout flare and subsequent cardiovascular events among patients with gout. JAMA.

[13] GBD 2019 Risk Factors Collaborators (2020). Global burden of 87 risk factors in 204 countries and territories, 1990–2019: a systematic analysis for the Global Burden of Disease Study 2019. Lancet.

[14] GBD 2019 Diseases and Injuries Collaborators (2020). Global burden of 369 diseases and injuries in 204 countries and territories, 1990–2019: a systematic analysis for the Global Burden of Disease Study 2019. Lancet.

[15] GBD 2019 Demographics Collaborators (2020). Global age-sex-specific fertility, mortality, healthy life expectancy (HALE), and population estimates in 204 countries and territories, 1950–2019: a comprehensive demographic analysis for the Global Burden of Disease Study 2019. Lancet.

[16] Wallace SL, Robinson H, Masi AT, Decker JL, McCarty DJ, Yü TF (1977). Preliminary criteria for the classification of the acute arthritis of primary gout. Arthritis Rheum.

[17] Edwards NL, Sundy JS, Forsythe A, Blume S, Pan F, Becker MA (2011). Work productivity loss due to flares in patients with chronic gout refractory to conventional therapy. J Med Econ.

[18] Terkeltaub RA, Furst DE, Bennett K, Kook KA, Crockett RS, Davis MW (2010). High versus low dosing of oral colchicine for early acute gout flare: twenty-four-hour outcome of the first multicenter, randomized, double-blind, placebo-controlled, parallel-group, dose-comparison colchicine study. Arthritis Rheum.

[19] Wang CC, Lien SB, Huang GS (2009). Arthroscopic elimination of monosodium urate deposition of the first metatarsophalangeal joint reduces the recurrence of gout. Arthroscopy.

[20] Yu KH, Luo SF (2003). Younger age of onset of gout in Taiwan. Rheumatology.

[21] Yu TF (1984). Diversity of clinical features in gouty arthritis. Semin Arthritis Rheum.

[22] Vollset SE, Goren E, Yuan C-W (2020). Fertility, mortality, migration, and population scenarios for 195 countries and territories from 2017 to 2100: a forecasting analysis for the Global Burden of Disease Study. Lancet.

[23] Das Gupta P (1978). A general method of decomposing a difference between two rates into several components. Demography.

[24] Guillén AG, Te Karu L, Singh JA, Dalbeth N (2020). Gender and ethnic inequities in gout burden and management. Rheum Dis Clin North Am.

[25] Ting K, Gill TK, Keen H, Tucker GR, Hill CL (2016). Prevalence and associations of gout and hyperuricaemia: results from an Australian population-based study. Intern Med J.

[26] Harrold LR, Etzel CJ, Gibofsky A (2017). Sex differences in gout characteristics: tailoring care for women and men. BMC Musculoskelet Disord.

[27] Te Karu L, Dalbeth N, Stamp L (2021). Inequities in people with gout: a focus on Māori (Indigenous People) of Aotearoa New Zealand. Ther Adv Musculoskelet Dis.

[28] GBD Chronic Kidney Disease Collaboration (2020). Global, regional, and national burden of chronic kidney disease, 1990–2017: a systematic analysis for the Global Burden of Disease Study 2017. Lancet.

[29] Evans PL, Prior JA, Belcher J, Mallen CD, Hay CA, Roddy E (2018). Obesity, hypertension and diuretic use as risk factors for incident gout: a systematic review and meta-analysis of cohort studies. Arthritis Res Ther.

[30] Li R, Yu K, Li C (2018). Dietary factors and risk of gout and hyperuricemia: a meta-analysis and systematic review. Asia Pac J Clin Nutr.

[31] Lin K, McCormick N, Yokose C (2023). Interactions between genetic risk and diet influencing risk of incident female gout: discovery and replication analysis of four prospective cohorts. Arthritis Rheumatol.

[32] Zhang Y, Yang R, Dove A (2022). Healthy lifestyle counteracts the risk effect of genetic factors on incident gout: a large population-based longitudinal study. BMC Med.

[33] Choi HK, Curhan G (2008). Soft drinks, fructose consumption, and the risk of gout in men: prospective cohort study. BMJ.

[34] Dehlin M, Jacobsson L, Roddy E (2020). Global epidemiology of gout: prevalence, incidence, treatment patterns and risk factors. Nat Rev Rheumatol.

[35] Perez-Ruiz F, Desideri G (2018). Improving adherence to gout therapy: an expert review. Ther Clin Risk Manag.

[36] Fields TR, Batterman A (2018). How can we improve disease education in people with gout?. Curr Rheumatol Rep.

[37] Bodirsky BL, Dietrich JP, Martinelli E (2020). The ongoing nutrition transition thwarts long-term targets for food security, public health and environmental protection. Sci Rep.

[38] GBD 2019 Diseases and Injuries Collaborators (2020). Global burden of 302 diseases and injuries in 204 countries and territories, 1990–2019: a systematic analysis for the Global Burden of Disease Study 2019. Lancet.

