# Correction to *Lancet Rheumatol* 2024; 6: e507–17

**DOI:** 10.1016/S2665-9913(24)00303-5

**Published:** 2024-09-27

*GBD 2021 Gout Collaborators. Global, regional, and national burden of gout, 1990–2020, and projections to 2050: a systematic analysis of the Global Burden of Disease Study 2021.* Lancet Rheumatol *2024; **6:** e507–17*—In this Article, the spelling of Mohamed A Saleh's name was incorrect and his affiliation should have been as follows: “College of Medicine, University of Sharjah, Sharjah, United Arab Emirates (Prof M A Saleh PhD); Faculty of Pharmacy, Mansoura University, Mansoura, Egypt (Prof M A Saleh PhD).” These corrections have been made as of September 27, 2024.

