# Supplementary material for: Global, regional, and national burden of gout, 1990–2020, and projections to 2050: a systematic analysis of the Global Burden of Disease Study 2021
Source: Lancet Rheumatol. 2024 Jul 9;6(8):e507–17. doi: 10.1016/S2665-9913(24)00117-6 (PMC11263476; doi:10.1016/S2665-9913(24)00117-6)
Supplement: Supplementary appendix [file mmc1.pdf]

THE LANCET
Rheumatology

Supplementary appendix

This appendix formed part of the original submission and has been peer reviewed. We post it as supplied by the authors.

Supplement to: GBD 2021 Gout Collaborators. Global, regional, and national burden of gout, 1990–2020, and projections to 2050: a systematic analysis of the Global Burden of Disease Study 2021. *Lancet Rheumatol* 2024; published online July 9. https://doi.org/10.1016/S2665-9913(24)00117-6.

Appendix to *Global, regional, and national burden of gout, 1990-2020, and projections to 2050: a systematic analysis of the Global Burden of Disease Study 2021*

	Page
**Methods**	
Section 1: Non-fatal data seeking	1
Section 2. Severity distribution meta-analysis	2
Section 3. Tables and figures	3
Supplemental Table S1: MR-BRT crosswalk adjustment factors for gout	3
Supplemental Table S2: Covariates	3
Supplemental Table S3: Severity distribution	3
Risk factors	4
Forecasting	4
**Results**	
Supplemental Table S4: Sources of gout data in countries and regions	4
Supplemental Table S5. Super-regional, regional, and national breakdown of locations with data sources for gout	5
Supplemental Table S6: National and subnational sources in countries with subnational estimation	5
Supplemental Table S7: Prevalence, years lived with disability (YLDs), age-standardised rates of prevalence, and YLDs per 100,000 in 2020, and percentage change between 1990 and 2020 for gout globally, by region and country	6
Supplemental Table S8: Gout years lived with disability percentage attributable to risk factors by region and sex	19
Supplemental Table S9: Forecast of gout age-standardised prevalence per 100,000 and total cases globally and by region, both sexes, 2020–2050.	20
Input data sources	21
Author contributions	28

1. METHODS

This appendix provides more detailed methodology and supplemental figures, tables, and results for "Global, regional, and national burden of gout, 1990–2020 and projections to 2050: a systematic analysis for the Global Burden of Disease Study 2021". Portions of this appendix have been adapted from Vos et al. References are provided for adapted sections.

Vos T, Lim S, Abbafati C, et al. Global burden of 369 diseases and injuries in 204 countries and territories, 1990–2019: a systematic analysis for the Global Burden of Disease Study 2019. *The Lancet* 2020; **396**(10258): 1204-22.

Section 1. Non-fatal data seeking

The last systematic review was conducted in GBD 2013 for studies published between 1980 and 2009 using the following search terms on MEDLINE, EMBASE, CINAHL, CAB Abstracts, WHO Library (WHOLIS), and OpenSIGLE. For prevalence and incidence, the following search terms were used: (gout* OR hyperuricemia) AND (prevalen* OR inciden* OR cross-sectional

OR cross sectional OR epidemiol* OR survey OR population-based OR population based OR population study OR population sample OR cohort OR follow-up OR follow up OR longitudinal OR regist*) AND (list of names of all GBD countries).

Exclusion criteria were:

- Sub-populations clearly not representative of the national population
- Not a population-based study
- Low sample size (less than 150)
- Review rather than original studies

For GBD 2019, 15 additional studies shared through the GBD Collaborator Network were added. In addition, data from USA claims data for 2000 and 2010–2014 by state and Taiwan (province of China) claims data from 2016 were included.

Section 2. Severity distribution meta-analysis

To calculate the severity distribution of gout, we used three studies on the distribution of the number of gout attacks per year and fitted a lognormal curve using a least squared differences method. In the absence of data on the proportion of gout cases who have chronic polyarticular gout, we assumed the proportion is equal to those who would have 52 attacks a year (ie, weekly) or more as implied by the lognormal curve. The average number of attacks was estimated from the lognormal fit: 5.66 (95% UI 5.14–6.18). From two studies we derived an average duration of attacks of 6.1 days (5.4–6.8) by simple averaging. The resulting proportion of time symptomatic for acute gout was taken as the multiplication of these two estimates divided by the number of days in a year: 9.4% (8.0–10.9).

Distribution of cases by frequency

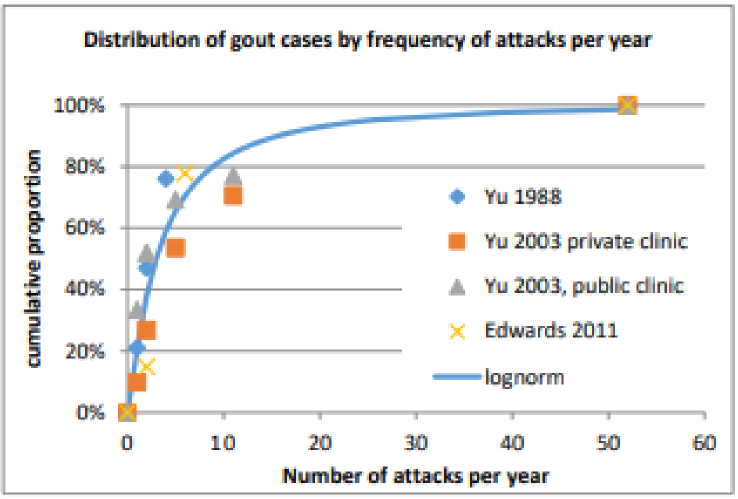

References

Edwards NL, Sundy JS, Forsythe A, Blume S, Pan F, Becker MA. Work productivity loss due to flares in patients with chronic gout refractory to conventional therapy. *Journal of Medical Economics*. 2011; 14(1):10-15.

Yu, KH, Luo SF, et al. Younger age of onset of gout in Taiwan. *Rheumatology*. 2003; 42(1):166-170.

Yu TF, et al. Diversity of clinical features in gouty arthritis. *Seminars in Arthritis and Rheumatism*. 1984; 13(4):360-368.

Section 3. Tables and figures

Table S1. MR-BRT[#] crosswalk adjustment factors for gout

Data input	Reference or alternative case definition	Gamma	Beta coefficient, log (95% CI)	Adjustment factor*
Physician-diagnosed gout	Ref	0.55	---	---
Self-reported gout	Alt		0.33 (0.050 to 0.60)	1.39 (1.05 to 1.83)
Gout identified with administrative data	Alt		0.29 (0.29 to 0.30)	1.34 (1.34 to 1.35)
USA claims data – 2000	Alt		–1.88 (–2.84 to –0.92)	0.15 (0.058 to 0.40)
USA claims data – 2016	Alt		–1.55 (–2.00 to –1.09)	0.22 (0.13 to 0.34)
Taiwan claims data – 2016	Alt		0.30 (0.27 to 0.33)	1.35 (1.31 to 1.40)

# MR-BRT=meta-regression—Bayesian, regularised, trimmed.

* Adjustment factor is the transformed Beta coefficient in normal space and can be interpreted as the factor by which the alternative case definition is adjusted to reflect what it would have been if measured as the reference.

Table S2. Covariates. Summary of covariates used in the gout DisMod-MR meta-regression

**Covariate**	**Type**	**Parameter**	**Exponentiated beta (95% uncertainty interval)**
Log-transformed age-standardised SEV scalar: gout	Country-level	Prevalence	3.48 (3.45–3.49)

Table S3. Severity distribution, details on the severity levels for gout in GBD 2019 and the associated disability weight (DW) with that severity

**Severity level**	**Lay description**	**DW (95% CI)**
Gout, acute	This person has severe pain and swelling in the leg making it very difficult to get up and down, stand, walk, lift, and carry heavy things. The person has trouble sleeping because of the pain.	0.295 (0.196–0.409)
Polyarticular gout (same as for severe RA)	This person has severe, constant pain and deformity in most joints, causing difficulty moving around, getting up and down, eating, dressing, lifting, carrying, and using the hands. The person often feels sadness, anxiety, and extreme fatigue.	0.581 (0.403–0.739)
Asymptomatic gout	This person has a diagnosis of gout without pain or functional difficulties.	0

Risk factors

High BMI is defined as greater than 20–25 kg/m^2 while kidney dysfunction is defined as estimated glomerular filtration rate less than 60 ml/min/1.73 m^2 or albumin to creatinine ratio greater than or equal to 30 mg/g.

Forecasting validation

Validation testing was conducted using estimates for osteoarthritis (OA) from 1990 to 2010 to project prevalence from 2010 to 2019 by age, sex, location, and year. The projections were then compared to the GBD OA prevalence results for this period by calculating the root mean squared error (RMSE) and bias (calculated as the median value of all predicted minus observed values by age, sex, location and year). Four tests were conducted: OA hip, OA knee, OA hand and OA other sites. In all the four tests the model RMSE was <0.0001.

2. RESULTS

Supplemental Table S4: Sources of gout data in countries and regions

Measure name	Total sources	Number of countries	Number of regions	Number of super- regions
-	131	35	15	7
Prevalence	114	34	15	7
Incidence	15	6	4	3
Relative risk	3	2	1	1
Standardised mortality ratio	1	1	1	1
Proportion	7	3	3	2

The data sources included in the model can be found here. https://ghdx.healthdata.org/gbd-2021/sources?components=5&causes=632&locations=214, and are listed in this appendix from page 23. The GBD model incorporated data containing all measures listed in Table S4.

Supplemental Table S5. Super-regional, regional, and national breakdown of locations with data sources for gout

Super-region	Region	Countries
Central Europe, eastern Europe, and central Asia	Central Asia	Georgia
	Central Europe	Czech Republic, Poland
High income	Australasia	Australia, New Zealand
	High income Asia Pacific	Singapore
	High income North America	Canada, USA
	Western Europe	Germany, Greece, Italy, Netherlands, Norway, Portugal, Spain, Sweden, United Kingdom
Latin America and Caribbean	Andean Latin America	Ecuador
	Caribbean	Cuba
	Central Latin America	Mexico
North Africa and Middle East	North Africa and Middle East	Iran, Kuwait, Lebanon, Türkiye
Southeast Asia, east Asia, and Oceania	Southeast Asia	Indonesia, Philippines, Thailand, Vietnam
	South Asia	Bangladesh, India, Pakistan
	East Asia	China, Taiwan
	Oceania	Samoa
Sub-Saharan Africa	Western sub-Saharan Africa	Nigeria

Supplemental Table S6: National and subnational sources of gout data in countries with subnational estimation

Location	National source counts	Subnational source counts	Total source counts
China	0	10	10
UK	22	7	29
Indonesia	1	0	1
India	0	5	5
Iran	0	1	1
Italy	1	1	2
Mexico	2	36	38
Nigeria	0	1	1
Norway	4	0	4
New Zealand	7	22	29
Pakistan	0	2	2
Philippines	1	1	2
Poland	0	48	48
Sweden	1	3	4
USA	13	820	833

Supplemental Table 7: Prevalence, years lived with disability (YLDs), age-standardised rates of prevalence, and YLDs per 100,000 in 2020, and percentage change between 1990 and 2020 for gout globally, regionally, and by country

Location	Number of prevalent cases (95% UI)	% change in number of prevalent cases from 1990 to 2020 (95% UI)	Age-standardised prevalence rate per 100 000 (95% UI)	% change in age-standardised prevalence rate from 1990 to 2020 (95% UI)	Number of YLDs (95% UI)	Age-standardised rate of YLDs per 100 000 in 2020 (95% UI)	% change in age-standardised rate of YLDs per 100 000 from 1990 to 2020 (95% UI)
GLOBAL	55 800 000 (44 400 000–69 800 000)	150.6 (142.7–159.2)	659·3 (525·4–822·3)	22·5 (20·9–24·2)	1 730 000 (1 220 000–2 390 000)	20·5 (14·4–28·2)	22·0 (19·8–24·2)
CENTRAL EUROPE, EASTERN EUROPE, AND CENTRAL ASIA	2 490 000 (1 940 000–3 150 000)	49.1 (45.1–52.8)	411·4 (319·8–518·3)	15·0 (13·6–16·3)	76 500 (53 400–105 000)	12·7 (8·8–17·4)	15·1 (12·0–18·0)
**Central Asia**	365 000 (283 000–468 000)	97.3 (89.7–103.9)	441·1 (340·4–550·0)	16·5 (13·1–19·3)	11 600 (7890–15 900)	13·8 (9·5–18·8)	16·5 (10·5–22·5)
Armenia	18 500 (14 100–22 700)	74.3 (62.1–87.2)	454·6 (353·0–562·2)	21·5 (15·2–27·6)	572 (396–804)	14·1 (9·6–19·7)	19·9 (3·4–36·0)
Azerbaijan	48 300 (37 600–63 300)	151.1 (133.5–164.4)	458·1 (353·1–580·9)	22·1 (13·7–28·0)	1550 (1080–2180)	14·5 (10·0–19·8)	23·4 (6·8–43·1)
Georgia	23 800 (18 500–29 600)	-1.8 (-8.1–4.2)	432·1 (340·0–536·9)	10·8 (4·6–16·4)	731 (518–1010)	13·4 (9·4–18·5)	9·0 (-3·7–26·5)
Kazakhstan	84 100 (65 500–109 000)	66.6 (56.7–78.8)	452·2 (354·8–570·4)	18·7 (11·7–26·2)	2640 (1770–3770)	14·0 (9·5–19·7)	17·9 (3·2–36·9)
Kyrgyzstan	21 500 (16 900–26 600)	89.1 (77.1–101.5)	416·3 (323·2–508·4)	13·2 (7·6–19·6)	680 (475–963)	13·0 (9·1–18·3)	12·5 (-1·6–29·4)
Mongolia	11 700 (9250–15 100)	172.0 (154.2–195.6)	417·2 (324·1–529·2)	9·5 (3·3–16·0)	372 (256–561)	13·0 (9·1–18·3)	9·6 (-2·7–21·8)
Tajikistan	24 000 (18 500–31 100)	131.7 (116.4–150.0)	389·2 (299·4–503·1)	11·7 (6·5–19·5)	765 (495–1100)	12·2 (8·1–17·2)	11·5 (-4·6–28·1)
Turkmenistan	20 300 (15 800–25 300)	157.8 (141.4–174.8)	463·2 (356·5–568·8)	21·2 (14·6–30·5)	641 (442–882)	14·5 (10·1–19·8)	20·1 (6·1–37·0)
Uzbekistan	112 000 (88 200–140 000)	173.9 (153.8–192.0)	445·8 (339·3–547·1)	17·5 (9·9–23·7)	3630 (2460–5050)	14·1 (9·8–19·2)	19·1 (3·6–34·6)
**Central Europe**	709 000 (551 000–911 000)	54.5 (48.6–60.7)	364·4 (287·1–461·8)	14·5 (12·2–16·2)	21 700 (15 100–30 000)	11·3 (7·9–15·4)	14·7 (9·5–18·8)
Albania	14 200 (10 900–18 200)	105.1 (86.4–125.0)	349·2 (273·7–440·4)	13·7 (6·2–21·5)	439 (306–627)	10·9 (7·6–15·7)	13·3 (-1·0–32·8)
Bosnia and Herzegovina	19 300 (14 700–24 800)	53.3 (39.0–69.0)	350·4 (268·7–444·3)	19·5 (13·2–25·6)	587 (390–805)	10·8 (7·4–14·8)	18·3 (2·4–41·6)

Bulgaria	45 200 (34 000–57 900)	15.6 (8.5–25.4)	354·7 (272·7–446·6)	7·4 (0·9–14·4)	1380 (959–1900)	11·0 (7·6–15·1)	8·3 (-4·7–24·8)
Croatia	28 000 (21 700–34 900)	44.0 (34.1–54.1)	364·3 (283·2–458·8)	17·3 (10·4–23·1)	854 (598–1190)	11·3 (7·9–15·8)	17·6 (2·6–34·5)
Czechia	69 000 (52 900–87 600)	68.2 (55.5–81.9)	367·3 (287·8–461·3)	18·0 (11·2–26·1)	2100 (1470–2950)	11·4 (8·1–15·8)	18·2 (-4·1–37·1)
Hungary	62 200 (47 700–80 200)	39.1 (32.6–47.5)	362·2 (284·5–458·9)	13·4 (7·9–20·1)	1910 (1340–2690)	11·3 (7·9–15·9)	15·1 (0·2–29·7)
Montenegro	3510 (2710–4390)	63.9 (53.4–75.9)	376·5 (291·7–471·7)	11·1 (4·3–18·2)	109 (74.9–146)	11·8 (8·1–15·6)	10·4 (-5·7–29·7)
North Macedonia	11 700 (8830–14 800)	89.4 (76.7–102.5)	373·2 (289·0–464·6)	15·4 (8·1–23·9)	364 (254–491)	11·7 (8·2–15·8)	15·6 (0·8–32·8)
Poland	238 000 (186 000–303 000)	70.3 (63.7–77.2)	369·3 (290·5–464·5)	13·9 (11·8–16·3)	7260 (5040–9990)	11·4 (7·9–15·4)	14·4 (10·3–20·5)
Romania	121 000 (94 500–155 000)	41.5 (33.3–51.4)	361·9 (285·0–460·0)	15·7 (9·6–22·3)	3700 (2510–5020)	11·2 (7·7–15·2)	14·7 (-1·5–31·4)
Serbia	52 900 (40 900–69 200)	50.0 (40.5–63.0)	369·2 (288·1–472·7)	14·6 (9·0–20·6)	1620 (1140–2300)	11·5 (8·0–16·4)	14·1 (0·6–29·8)
Slovakia	31 100 (23 700–38 600)	69.4 (59.5–79.0)	356·3 (279·3–442·1)	13·6 (6·5–19·0)	952 (677–1310)	11·0 (7·8–15·3)	13·8 (-0·8–31·2)
Slovenia	14 000 (10 700–17 400)	87.4 (75.1–101.6)	367·7 (285·9–459·6)	18·0 (11·4–23·5)	427 (298–590)	11·4 (7·9–15·8)	18·5 (1·8–33·9)
**Eastern Europe**	1 410 000 (1 110 000–1 780 000)	37.9 (33.9–42.2)	430·3 (334·5–541·2)	14·8 (13·1–16·5)	43 200 (30 300–59 600)	13·3 (9·2–18·2)	14·7 (10·9–18·1)
Belarus	60 400 (47 500–76 900)	34.7 (26.2–45.5)	401·1 (315·0–510·9)	13·2 (7·0–21·5)	1870 (1260–2650)	12·5 (8·5–17·6)	13·9 (-0·9–29·8)
Estonia	10 100 (8030–12 700)	39.6 (29.8–50.1)	442·6 (348·8–550·5)	20·8 (13·8–28·5)	310 (220–426)	13·8 (9·6–18·9)	20·8 (4·6–38·4)
Latvia	14 500 (11 000–18 400)	15.2 (7.8–25.5)	422·7 (327·8–540·7)	15·6 (9·3–23·5)	442 (321–613)	13·0 (9·1–18·0)	15·4 (2·0–31·4)
Lithuania	20 500 (16 000–25 700)	25.8 (18.3–35.4)	410·4 (321·5–510·0)	10·7 (4·7–17·7)	625 (433–846)	12·7 (8·8–17·5)	11·4 (-1·1–25·6)
Moldova	23 000 (18 100–28 500)	43.1 (33.2–54.6)	410·7 (324·1–508·4)	14·3 (7·4–22·0)	714 (508–1000)	12·8 (8·9–17·9)	14·5 (0·4–31·3)
Russia	988 000 (772 000–1 240 000)	48.6 (43.8–53.7)	437·6 (339·0–546·7)	17·2 (15·3–18·7)	30 100 (20 800–42 100)	13·5 (9·2–18·7)	17·1 (13·7–19·7)
Ukraine	297 000 (232 000–380 000)	12.8 (6.8–18.0)	416·4 (323·5–529·2)	8·3 (3·6–13·3)	9100 (6360–12 600)	12·9 (9·1–17·5)	8·5 (-5·3–23·4)

HIGH INCOME	18 700 000 (15 300 000–23 000 000)	132.3 (124.0–145.5)	1025·9 (845·9–1272·0)	44·3 (39·1–50·9)	570 000 (407 000–771 000)	31·7 (22·8–42·9)	43·2 (38·4–50·2)
**Australasia**	669 000 (520 000–873 000)	168.8 (150.2–194.1)	1424·4 (1129·6–1853·8)	32·3 (23·5–44·3)	20 400 (14 600–28 600)	43·9 (30·9–60·8)	31·8 (21·1–42·5)
Australia	551 000 (424 000–723 000)	180.9 (155.3–215.0)	1400·2 (1093·3–1831·8)	37·6 (25·6–53·8)	16 800 (12 000–23 800)	43·1 (30·3–60·2)	36·7 (23·1–51·7)
New Zealand	118 000 (94 900–146 000)	123.7 (108.8–141.6)	1547·4 (1272·5–1935·9)	12·6 (5·2–21·1)	3600 (2570–4890)	47·8 (33·9–65·1)	13·5 (5·4–23·7)
**High-income Asia Pacific**	2 700 000 (2 080 000–3 440 000)	105.6 (92.9–121.1)	728·4 (576·1–933·3)	13·1 (11·0–15·5)	83 500 (58 400–116 000)	22·9 (16·0–31·6)	13·3 (9·6–17·4)
Brunei	2960 (2330–3860)	238.9 (205.9–265.0)	760·7 (609·2–988·8)	14·0 (6·4–21·2)	94.8 (66.2–140)	23·8 (17·2–34·8)	14·0 (1·6–24·6)
Japan	1 980 000 (1 510 000–2 530 000)	81.8 (68.1–97.2)	723·9 (573·6–921·8)	11·4 (9·8–13·5)	61 000 (43 200–85 400)	22·9 (16·0–31·8)	11·8 (9·1–14·7)
South Korea	653 000 (502 000–841 000)	214.1 (185.4–243.3)	730·5 (572·4–942·3)	19·9 (12·7–27·8)	20 200 (14 000–28 600)	22·7 (15·7–31·5)	20·2 (8·9–37·4)
Singapore	67 700 (53 600–87 200)	313.8 (273.6–343.5)	806·4 (639·9–1026·0)	25·4 (17·6–34·1)	2140 (1430–2990)	25·5 (17·2–35·4)	25·4 (13·2–37·5)
**High-income North America**	9 680 000 (8 130 000–11 600 000)	199.6 (181.7–225.7)	1719·8 (1450·2–2078·7)	76·6 (65·0–90·7)	291 000 (210 000–394 000)	52·5 (37·2–70·6)	74·0 (61·7–87·3)
Canada	908 000 (716 000–1 130 000)	147.0 (128.6–165.6)	1494·0 (1199·0–1887·2)	28·8 (20·0–36·5)	27 900 (19 100–39 200)	46·6 (31·9–65·8)	28·0 (13·5–41·6)
Greenland	1050 (839–1350)	114.7 (92.8–137.0)	1459·9 (1183·3–1860·1)	27·9 (20·9–35·3)	32.6 (22.6–44.8)	45·3 (32·0–62·5)	27·1 (15·9–40·3)
USA	8 800 000 (7 430 000–10 500 000)	207.8 (187.8–236.6)	1746·2 (1476·4–2101·3)	83·0 (70·6–99·5)	264 000 (190 000–359 000)	53·1 (37·8–71·6)	80·2 (67·2–97·0)
**Southern Latin America**	759 000 (601 000–974 000)	118.1 (106.6–128.9)	926·0 (739·8–1196·5)	23·4 (17·9–29·2)	23 700 (16 100–33 100)	29·0 (19·9–40·6)	23·1 (13·7–31·8)
Argentina	479 000 (379 000–627 000)	101.7 (88.3–115.8)	904·4 (716·4–1189·5)	22·3 (14·8–30·5)	15 000 (10 100–21 400)	28·4 (19·1–40·7)	21·7 (10·9–35·4)
Chile	236 000 (186 000–296 000)	184.6 (163.4–208.8)	981·2 (782·0–1235·4)	24·4 (15·9–33·2)	7300 (5150–9890)	30·5 (21·3–41·2)	25·1 (14·1–41·1)
Uruguay	43 400 (33 800–52 500)	58.7 (49.7–67.3)	890·6 (701·7–1087·1)	20·2 (14·3–28·0)	1340 (905–1940)	27·8 (18·9–40·2)	19·4 (8·9–32·5)
**Western Europe**	4 920 000 (3 770 000–6 240 000)	68.5 (63.5–73.9)	627·3 (499·5–802·7)	14·6 (11·8–16·4)	151 000 (105 000–207 000)	19·6 (13·5–26·7)	14·7 (11·1–18·9)
Andorra	945 (736–1180)	161.3 (142.2–180.7)	668·4 (518·4–839·0)	7·1 (0·2–14·6)	29.4 (19.6–43.8)	20·9 (14·0–30·9)	7·0 (-6·6–19·5)

Austria	99 700 (78 900–127 000)	74.0 (62.6–83.7)	632·4 (505·5–808·5)	19·1 (11·4–25·7)	3 070 (2 080–4 190)	19·8 (13·3–27·2)	19·1 (5·6–34·6)
Belgium	124 000 (95 600–157 000)	59.9 (51.3–69.8)	627·1 (487·7–809·3)	14·4 (8·5–22·1)	3770 (2620–5150)	19·4 (13·2–26·3)	13·3 (1·9–31·0)
Cyprus	11 800 (9050–15 300)	163.6 (148.4–183.8)	609·9 (477·3–787·9)	11·5 (5·2–20·4)	366 (240–520)	19·1 (12·5–27·1)	10·9 (-4·2–26·4)
Denmark	63 500 (47 500–80 900)	59.2 (48.1–67.6)	628·0 (494·5–794·8)	16·1 (9·2–22·1)	1970 (1310–2790)	19·7 (13·7–27·7)	16·1 (3·1–31·4)
Finland	64 300 (49 800–82 100)	79.9 (64.1–96.8)	623·0 (494·8–795·5)	17·6 (11·6–25·5)	1980 (1350–2760)	19·5 (13·2–27·0)	18·3 (5·0–30·9)
France	713 000 (553 000–901 000)	75.0 (63.7–87.5)	614·2 (493·8–788·4)	15·1 (8·5–22·1)	21 900 (15 200–30 600)	19·2 (13·5–26·8)	15·0 (2·9–31·3)
Germany	1 040 000 (798 000–1 330 000)	63.3 (51.2–74.4)	643·7 (511·5–834·8)	17·9 (9·6–24·7)	32 000 (22 000–44 900)	20·1 (13·8–28·4)	17·2 (3·0–31·4)
Greece	136 000 (104 000–170 000)	51.4 (42.0–63.7)	686·9 (544·3–873·1)	9·9 (3·6–16·6)	4190 (2940–5850)	21·5 (14·7–30·0)	10·2 (-4·1–22·9)
Iceland	3380 (2660–4300)	111.8 (95.8–127.7)	657·1 (518·3–830·8)	12·1 (4·3–20·7)	106 (72.9–147)	20·7 (14·1–28·6)	12·5 (-1·2–25·8)
Ireland	47 100 (36 600–60 200)	107.9 (94.0–120.8)	657·7 (519·3–836·0)	12·8 (5·0–20·4)	1470 (976–2060)	20·7 (13·9–29·1)	13·3 (-3·1–27·6)
Israel	72 300 (56 600–91 100)	165.8 (151.7–183.1)	651·6 (514·5–818·1)	12·7 (6·5–20·2)	2260 (1490–3230)	20·5 (13·6–29·2)	11·9 (-0·3–26·8)
Italy	669 000 (516 000–840 000)	61.6 (55.6–68.8)	557·3 (446·7–711·3)	11·0 (8·4–13·4)	20 500 (14 500–28 200)	17·4 (12·2–24·1)	11·9 (7·8–16·2)
Luxembourg	6180 (4910–7730)	113.6 (100.9–125.7)	641·4 (505·9–812·3)	15·3 (9·1–21·7)	192 (136–272)	20·1 (14·2–28·5)	15·3 (1·7–28·5)
Malta	5130 (3930–6580)	122.3 (102.0–142.5)	630·6 (501·4–803·0)	16·8 (10·2–23·2)	160 (108–223)	19·9 (13·3–27·8)	16·6 (3·7–30·2)
Monaco	529 (406–661)	51.5 (42.5–60.8)	662·1 (521·2–826·2)	12·4 (6·3–19·1)	16.3 (11.1–22.4)	20·8 (14·3–28·5)	12·1 (0·8–26·9)
Netherlands	204 000 (155 000–258 000)	85.2 (74.4–99.6)	673·3 (518·7–866·0)	15·7 (9·2–23·5)	6330 (4200–8650)	21·2 (14·1–28·7)	14·9 (-2·9–30·8)
Norway	48 500 (37 500–61 500)	64.5 (59.3–70.1)	560·9 (446·7–712·0)	14·0 (11·3–16·5)	1500 (1020–2090)	17·5 (12·1–24·3)	14·3 (8·0–20·2)
Portugal	128 000 (99 300–162 000)	77.1 (66.2–93.4)	624·5 (499·3–796·8)	14·5 (8·9–22·4)	3950 (2640–5580)	19·6 (13·0–28·3)	15·6 (4·8–31·4)
San Marino	364 (281–456)	99.4 (87.2–115.4)	640·4 (508·8–802·2)	9·0 (2·2–17·1)	11.2 (7.87–15.4)	20·1 (13·7–27·5)	8·8 (-1·7–23·8)

Spain	534 000 (418 000–685 000)	84.5 (72.7–100.5)	638·7 (509·2–828·5)	12·7 (4·9–20·0)	16 600 (11 300–23 400)	20·1 (13·5–28·7)	13·6 (0·2–27·0)
Sweden	110 000 (83 100–144 000)	46.5 (38.3–55.1)	615·1 (477·6–815·5)	8·6 (3·2–14·5)	3390 (2350–4660)	19·2 (13·1–26·9)	8·7 (-2·5–20·3)
Switzerland	95 900 (75 400–123 000)	80.6 (67.9–94.2)	622·6 (495·9–802·2)	12·3 (5·5–20·9)	2950 (2030–4250)	19·4 (13·3–28·0)	13·2 (-1·9–30·2)
UK	736 000 (568 000–931 000)	57.8 (54.6–61.3)	662·6 (529·4–838·4)	15·8 (14·0–17·5)	22 700 (15 600–31 200)	20·7 (14·2–28·7)	15·3 (13·2–17·5)
LATIN AMERICA AND CARIBBEAN	1 430 000 (1 130 000–1 780 000)	204.1 (190.4–218.6)	230·6 (183·2–285·2)	24·4 (23·2–26·1)	45 100 (31 100–61 500)	7·2 (5·0–9·9)	23·6 (19·5–26·8)
**Andean Latin America**	175 000 (138 000–221 000)	238.8 (224.5–256.0)	289·6 (229·6–365·5)	30·4 (25·1–37·4)	5530 (3590–7580)	9·1 (6·0–12·5)	28·6 (15·5–42·1)
Bolivia	27 900 (21 500–35 100)	256.3 (230.0–281.8)	282·7 (221·8–351·0)	33·4 (24·5–41·4)	886 (589–1230)	8·9 (5·9–12·2)	32·2 (14·0–54·3)
Ecuador	50 700 (39 500–62 500)	252.1 (229.3–276.3)	311·1 (244·9–381·0)	29·4 (22·2–37·6)	1590 (1060–2200)	9·7 (6·5–13·4)	26·0 (9·0–46·3)
Peru	96 500 (77 200–123 000)	230.6 (208.7–248.2)	281·3 (223·9–359·9)	30·4 (23·6–37·9)	3050 (1980–4270)	8·9 (5·7–12·5)	29·0 (9·3–49·5)
**Caribbean**	129 000 (104 000–157 000)	137.6 (126.8–148.0)	245·7 (198·1–300·8)	24·4 (21·0–27·8)	4060 (2850–5560)	7·8 (5·4–10·6)	23·4 (14·5–34·9)
Antigua and Barbuda	265 (207–331)	168.0 (147.0–188.5)	248·7 (197·5–308·5)	31·0 (23·6–37·5)	8.29 (5.55–11.8)	7·8 (5·2–11·0)	28·7 (5·4–48·9)
The Bahamas	1070 (841–1310)	190.1 (164.5–213.7)	252·8 (200·0–306·2)	23·9 (16·7–31·4)	33.8 (22.5–47.1)	7·9 (5·4–10·9)	20·3 (0·6–43·5)
Barbados	1140 (898–1400)	109.5 (90.1–128.4)	254·8 (204·4–309·5)	25·8 (19·7–33·1)	35.9 (24.6–51)	8·1 (5·8–11·2)	25·5 (8·7–45·8)
Belize	886 (709–1100)	329.3 (303.6–360.4)	268·4 (212·8–327·0)	33·6 (26·3–41·6)	28.2 (18.9–40)	8·4 (5·7–11·8)	29·7 (13·2–52·1)
Bermuda	301 (237–372)	112.2 (92.4–135.6)	270·9 (218·9–335·3)	25·2 (18·4–33·0)	9.31 (6.83–12.8)	8·5 (6·1–11·8)	22·9 (7·6–48·4)
Cuba	44 000 (35 700–54 600)	108.9 (93.6–123.9)	252·8 (204·8–310·2)	25·1 (19·5–31·8)	1390 (965–1960)	8·0 (5·5–11·0)	24·9 (6·9–46·1)
Dominica	227 (181–281)	74.1 (64.6–85.2)	265·5 (212·0–327·0)	32·7 (25·8–39·9)	7.14 (5.19–9.7)	8·4 (6·0–11·6)	31·8 (14·6–55·9)
Dominican Republic	24 500 (19 800–30 100)	208.3 (191.1–229.7)	242·2 (198·4–297·6)	30·9 (23·8–38·1)	776 (545–1080)	7·6 (5·4–10·5)	29·7 (9·3–54·6)
Grenada	291 (225–362)	131.5 (114.0–151.7)	251·7 (198·0–307·7)	36·3 (29·5–44·1)	9.18 (6.14–12.5)	7·9 (5·3–10·7)	32·3 (13·0–55·2)

Guyana	1670<br>(1310–2070)	95.0<br>(80.8–113.0)	242·6<br>(191·3–294·7)	28·3<br>(21·9–35·5)	52.6<br>(36.3–75.8)	7·6<br>(5·2–10·6)	27·3<br>(9·2–45·0)
Haiti	17 700<br>(13 800–21 700)	176.2<br>(161.2–195.4)	205·3<br>(162·7–254·1)	19·1<br>(13·3–26·1)	569<br>(375–807)	6·5<br>(4·5–8·9)	19·3<br>(-0·1–39·8)
Jamaica	7960<br>(6390–9960)	125.0<br>(108.9–137.5)	259·5<br>(207·1–326·7)	30·1<br>(22·7–36·6)	251<br>(178–335)	8·2<br>(5·7–11·0)	27·5<br>(9·3–46·5)
Puerto Rico	16 400<br>(12 600–20 200)	107.7<br>(90.0–128.9)	280·6<br>(222·9–344·2)	27·4<br>(20·2–34·4)	502<br>(349–699)	8·8<br>(5·8–12·1)	24·3<br>(8·0–51·9)
Saint Kitts and Nevis	186<br>(145–232)	169.3<br>(143.5–201.1)	260·3<br>(206·5–320·8)	28·5<br>(23·0–35·5)	5.91<br>(4.12–8.61)	8·2<br>(5·6–11·6)	27·6<br>(9·0–48·3)
Saint Lucia	558<br>(436–701)	218.7<br>(199.9–242.9)	253·3<br>(199·2–316·7)	32·3<br>(24·1–39·6)	17.6<br>(12.4–25)	8·0<br>(5·7–11·4)	30·2<br>(7·9–51·7)
Saint Vincent and the Grenadines	342<br>(270–423)	150.3<br>(127.5–172.9)	253·0<br>(202·4–311·0)	36·2<br>(27·0–42·9)	10.8<br>(7.26–15.4)	8·0<br>(5·3–11·3)	33·9<br>(14·8–58·2)
Suriname	1540<br>(1210–1880)	177.2<br>(159.1–205.2)	245·1<br>(194·8–295·2)	25·3<br>(19·3–35·1)	48.9<br>(33–68.6)	7·8<br>(5·3–10·8)	24·5<br>(6·9–46·0)
Trinidad and Tobago	4830<br>(3810–5970)	153.8<br>(133.8–174.4)	265·0<br>(209·0–323·3)	27·4<br>(19·7–35·0)	151<br>(102–219)	8·3<br>(5·5–11·9)	26·4<br>(7·6–48·3)
Virgin Islands	437<br>(338–557)	116.2<br>(91.1–142.1)	274·6<br>(218·2–339·1)	29·1<br>(21·2–35·8)	13.4<br>(9.42–18.9)	8·5<br>(6·0–11·9)	27·2<br>(10·4–51·7)
**Central Latin America**	478 000<br>(378 000–589 000)	205.8<br>(189.1–223.3)	188·4<br>(148·6–231·1)	21·4<br>(19·7–23·3)	15 300<br>(10 400–21 200)	6·0<br>(4·1–8·3)	21·1<br>(15·4–27·2)
Colombia	92 400<br>(74 200–113 000)	222.3<br>(197.0–265.1)	171·1<br>(136·7–210·2)	29·1<br>(21·9–35·8)	2 60<br>(2 80–4110)	5·5<br>(3·9–7·6)	29·0<br>(7·4–53·5)
Costa Rica	9780<br>(7760–11 900)	221.9<br>(196.1–249.6)	182·8<br>(145·1–223·0)	24·2<br>(17·7–31·6)	311<br>(212–416)	5·8<br>(4·0–7·8)	21·2<br>(1·5–40·4)
El Salvador	10 500<br>(8330–12 800)	137.2<br>(123.7–153.3)	172·2<br>(135·5–211·8)	29·8<br>(22·0–37·4)	335<br>(227–467)	5·5<br>(3·7–7·6)	29·2<br>(6·0–50·9)
Guatemala	21 500<br>(17 000–26 200)	251.7<br>(229.4–280.7)	160·3<br>(127·7–199·7)	29·5<br>(20·8–38·3)	687<br>(445–972)	5·1<br>(3·4–7·1)	28·6<br>(9·8–53·6)
Honduras	12 000<br>(9550–14 900)	279.0<br>(251.6–303.2)	164·2<br>(131·5–200·1)	28·7<br>(21·0–36·8)	391<br>(269–564)	5·3<br>(3·6–7·3)	28·8<br>(8·1–53·2)
Mexico	262 000<br>(206 000–324 000)	190.3<br>(175.6–208.3)	205·8<br>(161·4–253·7)	16·7<br>(14·6–18·7)	8350<br>(5670–11 500)	6·5<br>(4·5–8·9)	17·1<br>(13·0–22·1)
Nicaragua	9440<br>(7440–11 800)	271.1<br>(247.7–295.8)	174·7<br>(139·7–213·4)	33·4<br>(26·0–41·8)	304<br>(208–408)	5·5<br>(3·9–7·4)	32·8<br>(10·5–55·1)
Panama	7640<br>(6010–9350)	244.3<br>(217.5–270.1)	176·7<br>(139·0–216·9)	36·4<br>(28·8–44·7)	243<br>(165–338)	5·6<br>(3·8–7·8)	33·3<br>(8·7–64·7)

Venezuela	53 200 (42 100–64 600)	220.7 (194.1–250.0)	173·0 (137·3–209·5)	25·8 (19·1–33·6)	1690 (1120–2450)	5·5 (3·7–7·9)	23·1 (5·9–49·8)
**Tropical Latin America**	648 000 (513 000–812 000)	211.4 (198.2–228.4)	254·7 (203·0–316·5)	26·4 (24·1–28·9)	20 200 (14 200–27 400)	7·9 (5·6–10·7)	25·5 (18·2–32·0)
Brazil	633 000 (501 000–794 000)	168.0 (147.0–188.5)	254·9 (203·1–317·0)	26·4 (24·1–28·9)	19 800 (13 900–26 800)	7·9 (5·6–10·7)	25·5 (18·0–32·2)
Paraguay	15 100 (12 200–18 500)	211.4 (195.7–228.6)	244·1 (197·1–299·3)	25·8 (19·4–31·8)	482 (327–704)	7·7 (5·3–11·3)	25·0 (6·7–49·2)
NORTH AFRICA AND MIDDLE EAST	2 610 000 (2 050 000–3 310 000)	218.1 (210.5–225.8)	525·9 (411·5–657·3)	20·1 (18·1–22·2)	81 800 (56 100–114 000)	16·2 (11·4–22·2)	19·2 (14·6–23·0)
Afghanistan	69 200 (52 500–86 300)	148.5 (124.9–178.1)	433·9 (339·4–529·0)	11·4 (5·5–16·3)	2150 (1330–3050)	13·0 (8·9–18·2)	8·7 (-2·7–21·5)
Algeria	195 000 (150 000–245 000)	245.5 (217.5–273.4)	523·0 (398·2–647·7)	20·7 (12·1–29·0)	6120 (4150–8400)	16·2 (11·4–21·9)	20·3 (6·1–35·8)
Bahrain	9620 (7280–12 500)	603.8 (518.5–691.1)	620·2 (482·6–762·8)	20·9 (13·8–28·9)	303 (192–428)	19·0 (12·7–25·0)	19·2 (5·0–38·1)
Egypt	404 000 (318 000–509 000)	182.5 (167.3–200.9)	571·0 (455·2–703·5)	27·5 (20·9–36·7)	12 700 (8860–18 400)	17·6 (12·4–25·3)	26·6 (14·0–39·9)
Iran	410 000 (322 000–519 000)	220.9 (210.0–234.7)	500·3 (389·5–636·2)	13·0 (11·0–15·3)	12 800 (8790–17 700)	15·4 (10·7–21·3)	12·1 (9·0–15·9)
Iraq	142 000 (110 000–175 000)	252.4 (227.9–274.1)	519·9 (398·7–636·2)	9·7 (3·3–15·8)	4370 (2930–6070)	15·7 (10·7–21·3)	7·5 (-5·0–22·7)
Jordan	48 800 (38 700–61 300)	548.0 (513.7–591.0)	589·9 (459·2–726·2)	22·9 (16·0–31·0)	1550 (1090–2190)	18·2 (13·3–25·8)	22·0 (9·9–37·0)
Kuwait	24 600 (19 500–31 700)	376.6 (340.9–412.4)	642·8 (502·9–783·3)	12·4 (6·3–19·4)	775 (530–1080)	19·7 (13·8–26·9)	9·6 (-3·3–25·0)
Lebanon	27 200 (21 200–33 700)	160.9 (144.2–181.8)	505·2 (394·9–626·1)	14·8 (7·9–21·4)	838 (589–1140)	15·5 (11·0–21·0)	14·0 (-1·9–29·3)
Libya	32 800 (26 200–41 400)	229.9 (209.9–252.2)	540·2 (416·2–671·8)	12·5 (6·1–20·1)	1020 (691–1430)	16·5 (11·7–22·6)	10·1 (-3·5–27·8)
Morocco	166 000 (129 000–215 000)	176.7 (152.1–198.7)	491·1 (383·1–627·7)	20·4 (11·9–29·9)	5150 (3620–7390)	15·0 (10·6–21·2)	17·9 (4·3–34·2)
Oman	17 600 (13 500–22 000)	331.1 (297.5–359.8)	590·7 (458·6–733·4)	35·2 (26·0–43·6)	571 (372–814)	18·3 (12·7–25·9)	35·9 (18·1–53·9)
Palestine	14 100 (11 100–17 800)	263.9 (239.0–289.0)	491·8 (381·5–600·0)	17·8 (11·8–24·1)	444 (303–614)	15·1 (10·4–20·6)	16·8 (2·2–33·7)
Qatar	15 600 (12 500–19 900)	1016.3 (926.7–1116.8)	763·1 (589·8–956·0)	25·6 (17·4–34·4)	502 (327–714)	23·4 (17·1–32·3)	24·6 (5·1–43·7)

Saudi Arabia	174 000 (136 000–221 000)	382.7 (348.8–419.1)	651·3 (507·8–806·9)	32·9 (24·9–41·7)	5520 (3890–7970)	19·9 (13·9–26·9)	32·1 (16·3–53·5)
Sudan	106 000 (82 700–134 000)	168.4 (145.6–186.3)	485·6 (377·0–610·4)	23·2 (13·8–31·3)	3330 (2320–4840)	14·9 (10·5–21·5)	22·2 (4·0–38·3)
Syria	67 100 (52 200–84 400)	156.8 (136.7–175.3)	508·6 (395·2–629·4)	12·7 (7·1–19·0)	2100 (1460–2970)	15·7 (11·1–21·8)	12·3 (1·4–28·3)
Tunisia	66 500 (51 900–84 700)	184.3 (164.9–209.7)	500·5 (393·4–631·8)	14·0 (7·4–22·6)	2050 (1410–2970)	15·3 (10·7–21·8)	11·8 (-2·1–29·0)
Türkiye	485 000 (380 000–618 000)	185.0 (165.8–207.3)	518·8 (405·9–653·8)	18·1 (10·1–26·3)	15 200 (10 500–21 100)	16·2 (11·2–22·4)	18·3 (3·0–32·6)
United Arab Emirates	64 300 (48 100–83 100)	1165.1 (1024.9–1286.4)	755·6 (597·3–959·6)	30·1 (21·9–38·6)	2080 (1 300–3 050)	23·2 (16·2–31·9)	29·5 (11·1–55·4)
Yemen	66 100 (52 700–83 000)	228.3 (208.4–247.8)	412·4 (321·0–508·9)	14·5 (8·1–21·2)	2 090 (1490–2950)	12·7 (9·2–18·3)	13·6 (-3·2–31·6)
SOUTH ASIA	6 410 000 (5 000 000–8 170 000)	163.9 (154.5–173.5)	421·2 (328·6–528·9)	7·8 (6·4–9·4)	199 000 (138 000–275 000)	12·9 (9·1–17·9)	8·8 (5·4–12·0)
Bangladesh	563 000 (434 000–702 000)	191.1 (172.1–212.1)	399·2 (307·3–491·3)	6·2 (0·2–12·7)	17 700 (12 400–24 200)	12·4 (8·8–16·7)	7·9 (-5·2–24·5)
Bhutan	2700 (2130–3450)	156.1 (135.7–173.9)	436·2 (340·0–555·1)	17·8 (11·4–24·4)	85.3 (58.1–121)	13·6 (9·4–19·1)	17·2 (5·5–28·3)
India	5 210 000 (4 070 000–6 630 000)	163.7 (153.3–175.1)	424·2 (332·4–533·3)	6·7 (5·5–8·2)	162 000 (112 000–224 000)	13·0 (9·2–18·0)	7·9 (4·8–11·2)
Nepal	95 700 (73 100–125 000)	154.0 (136.2–172.8)	401·2 (306·6–513·3)	9·7 (4·2–16·1)	2950 (2120–4100)	12·2 (8·8–16·8)	10·1 (-2·6–25·5)
Pakistan	535 000 (411 000–688 000)	143.1 (132.4–154.0)	421·9 (321·9–531·5)	16·1 (11·6–21·4)	16 800 (11 800–23 900)	12·9 (9·1–18·6)	15·9 (5·1–28·8)
SOUTHEAST ASIA, EAST ASIA, AND OCEANIA	21 700 000 (16 800 000–27 600 000)	177.5 (161.0–193.3)	774·2 (606·4–972·2)	25·1 (22·4–27·3)	682 000 (466 000–947 000)	24·3 (16·8–33·5)	24·8 (21·0–27·7)
**East Asia**	17 300 000 (13 300 000–21 900 000)	175.9 (157.2–194.1)	817·4 (641·2–1023·9)	26·4 (23·4–29·1)	542 000 (372 000–754 000)	25·7 (17·8–35·5)	26·0 (21·6–29·4)
China	16 600 000 (12 800 000–21 100 000)	177.2 (157.7–195.3)	813·5 (637·0–1019·2)	26·7 (23·5–29·3)	521 000 (358 000–726 000)	25·6 (17·7–35·3)	26·3 (21·6–29·9)
North Korea	262 000 (208 000–327 000)	114.3 (100.1–128.8)	791·3 (632·0–985·0)	14·6 (9·0–20·9)	8360 (5680–11 900)	25·2 (17·1–35·9)	15·4 (4·2–30·8)
Taiwan (province of China)	399 000 (319 000–499 000)	171.4 (149.5–197.6)	1049·3 (842·1–1313·0)	24·3 (14·2–36·6)	12 500 (8780–17 100)	33·2 (22·9–44·5)	24·0 (11·0–42·6)
**Oceania**	59 500 (46 700–76 500)	166.6 (154.7–178.1)	701·5 (555·1–888·3)	8·0 (3·9–12·4)	1870 (1290–2730)	21·6 (15·4–30·3)	7·5 (-1·6–16·9)

American Samoa	463<br>(362–599)	103.3<br>(88.5–119.1)	919·2<br>(730·7–1185·3)	7·8<br>(2·7–15·1)	14.3<br>(9.85–20)	28·2<br>(19·8–38·6)	6·7<br>(-4·7–20·0)
Cook Islands	215<br>(169–273)	101.9<br>(86.3–118.2)	883·7<br>(700·4–1125·2)	12·7<br>(6·3–19·5)	6.61<br>(4.7–9.76)	27·3<br>(19·3–39·8)	11·5<br>(3·3–22·4)
Fiji	6820<br>(5310–8880)	119.7<br>(101.4–137.4)	826·2<br>(647·8–1050·8)	14·9<br>(6·0–23·3)	213<br>(145–301)	25·5<br>(17·3–35·8)	14·6<br>(1·3–31·6)
Guam	1620<br>(1280–2020)	135.0<br>(110.8–154.4)	846·4<br>(668·3–1050·9)	12·5<br>(5·5–20·7)	51.1<br>(34.9–72)	26·7<br>(18·4–36·9)	13·1<br>(1·4–25·3)
Kiribati	561<br>(440–705)	106.9<br>(95.3–121.1)	696·9<br>(554·9–863·9)	7·2<br>(1·4–13·0)	17.7<br>(11.5–25.1)	21·5<br>(14·5–30·0)	7·8<br>(-2·4–20·7)
Marshall Islands	302<br>(234–393)	146.0<br>(127.0–163.4)	742·8<br>(585·1–940·9)	16·2<br>(9·2–22·7)	9.5<br>(6.43–13.4)	22·9<br>(15·7–30·8)	15·2<br>(2·0–32·0)
Federated States of Micronesia	629<br>(496–783)	71.0<br>(59.9–87.1)	792·1<br>(626·3–971·8)	11·8<br>(4·3–21·4)	20<br>(13.5–29.1)	24·6<br>(17·1–34·8)	11·7<br>(-1·8–29·5)
Nauru	42.8<br>(32.7–54.6)	19.2<br>(10.3–26.8)	770·4<br>(608·4–968·7)	2·8<br>(-3·9–8·3)	1.36<br>(0.883–1.87)	23·8<br>(16·8–31·4)	3·0<br>(-7·2–15·9)
Niue	17.7<br>(13.9–22.2)	18.9<br>(10.6–28.3)	837·2<br>(662·5–1044·3)	18·7<br>(12·3–27·2)	0.546<br>(0.373–0.743)	25·9<br>(17·8–35·8)	17·6<br>(4·1–30·4)
Northern Mariana Islands	517<br>(394–671)	109.0<br>(75.1–146.8)	904·7<br>(705·5–1149·1)	-0·9<br>(-8·2–6·3)	16.2<br>(11–23.3)	28·2<br>(20·4–38·6)	-1·8<br>(-13·0–8·9)
Palau	207<br>(162–274)	150.6<br>(128.0–173.9)	884·0<br>(695·2–1129·9)	16·6<br>(9·5–23·3)	6.47<br>(4.33–8.9)	27·4<br>(18·2–36·7)	15·5<br>(0·2–30·0)
Papua New Guinea	39 200<br>(30 900–50 000)	198.7<br>(181.2–216.0)	662·3<br>(524·1–837·2)	8·0<br>(2·4–14·3)	1240<br>(821–1840)	20·4<br>(14·3–29·2)	7·8<br>(-5·7–20·3)
Samoa	1320<br>(1040–1710)	84.7<br>(72.4–96.8)	831·1<br>(652·7–1091·7)	9·1<br>(2·7–16·2)	41.4<br>(28.5–57.7)	25·8<br>(18·1–35·8)	8·6<br>(-1·5–25·6)
Solomon Islands	2640<br>(2060–3500)	160.3<br>(140.4–178.2)	712·1<br>(560·0–916·2)	6·9<br>(-0·1–14·5)	83.8<br>(57.6–117)	22·0<br>(15·6–29·7)	5·5<br>(-5·6–17·2)
Tokelau	11.1<br>(8.54–13.9)	32.0<br>(23.6–39.7)	806·4<br>(627·6–1004·9)	26·0<br>(19·4–32·3)	0.345<br>(0.233–0.471)	25·0<br>(16·9–34·2)	26·4<br>(14·3–37·4)
Tonga	672<br>(527–857)	58.5<br>(48.6–70.9)	820·6<br>(641·1–1046·9)	13·4<br>(6·6–20·3)	21<br>(14.7–29.8)	25·6<br>(18·1–36·0)	13·1<br>(2·5–25·0)
Tuvalu	79.8<br>(62.3–99.2)	86.1<br>(74.6–98.8)	748·7<br>(595·1–916·1)	25·5<br>(17·9–33·9)	2.51<br>(1.77–3.42)	23·4<br>(16·7–31·5)	25·6<br>(8·6–45·1)
Vanuatu	1450<br>(1110–1880)	186.6<br>(164.6–205.1)	746·4<br>(570·2–951·3)	10·1<br>(2·7–18·0)	45.5<br>(31.7–64)	23·2<br>(15·9–32·0)	10·1<br>(-3·4–23·9)
**Southeast Asia**	4 350 000<br>(3 390 000–5 600 000)	184.5<br>(172.5–192.9)	642·1<br>(501·3–816·6)	21·3<br>(18·9–23·4)	138 000<br>(92 700–190 000)	20·2<br>(13·8–27·8)	22·0<br>(17·7–26·3)

Cambodia	76 200 (60 400–95 200)	211.8 (193.7–232.3)	559·7 (442·1–699·3)	16·4 (9·3–22·6)	2410 (1550–3350)	17·5 (11·3–23·8)	16·7 (1·2–28·7)
Indonesia	1 640 000 (1 280 000–2 120 000)	171.9 (160.5–179.7)	657·7 (513·7–829·3)	23·0 (19·8–25·8)	52 400 (35 500–72 800)	20·7 (14·4–28·5)	23·4 (19·0–28·6)
Laos	32 900 (26 000–42 900)	178.7 (157.9–200.7)	630·8 (498·2–810·8)	22·7 (13·8–30·7)	1060 (727–1550)	19·9 (13·9–28·8)	24·3 (9·0–41·3)
Malaysia	216 000 (168 000–271 000)	246.6 (220.0–271.2)	726·2 (565·0–901·8)	25·2 (18·9–33·0)	6790 (4620–9850)	22·6 (15·7–32·4)	25·3 (10·9–37·3)
Maldives	3260 (2550–4110)	434.9 (382.9–497.8)	736·2 (579·6–927·3)	23·5 (14·9–33·3)	105 (72.7–149)	23·2 (16·4–31·9)	23·9 (10·5–40·2)
Mauritius	12 700 (9790–15 800)	164.4 (140.3–190.9)	719·4 (561·6–890·3)	23·0 (14·7–31·0)	395 (267–568)	22·3 (14·9–31·7)	22·9 (8·4–36·3)
Myanmar	301 000 (236 000–364 000)	131.6 (116.4–146.2)	589·1 (457·6–710·0)	15·3 (7·7–21·1)	9570 (6530–13 500)	18·5 (12·9–26·0)	16·1 (2·9–32·9)
Philippines	556 000 (431 000–709 000)	217.1 (205.5–226.4)	624·0 (486·6–783·3)	22·9 (21·0–24·9)	17 600 (11 700–24 600)	19·5 (13·3–27·2)	23·7 (20·2–27·0)
Seychelles	883 (690–1130)	162.8 (145.7–185.5)	731·8 (574·9–934·5)	24·2 (16·9–32·8)	27.8 (18.5–40.1)	22·8 (15·5–32·4)	23·4 (10·5–41·8)
Sri Lanka	168 000 (131 000–211 000)	143.5 (125.8–163.2)	640·9 (507·5–793·4)	13·1 (6·3–20·7)	5210 (3580–7080)	19·9 (13·8–26·7)	12·8 (0·5–26·5)
Thailand	728 000 (570 000–949 000)	207.7 (178.2–240.3)	700·7 (554·6–905·2)	21·1 (12·5–29·2)	22 700 (15 700–31 700)	21·9 (15·2–30·5)	22·4 (8·9–36·3)
Timor-Leste	5190 (4020–6630)	165.6 (142.8–192.0)	591·3 (462·9–738·2)	11·1 (4·9–16·9)	163 (109–225)	18·3 (12·6–25·2)	11·0 (-4·8–24·2)
Viet Nam	601 000 (471 000–786 000)	192.2 (172.3–215.3)	573·4 (444·7–738·7)	19·0 (12·8–26·8)	19 300 (12 800–26 700)	18·2 (12·0–24·6)	20·2 (5·4–34·9)
SUB-SAHARAN AFRICA	2 470 000 (1 940 000–3 140 000)	144.9 (140.7–149.4)	455·9 (354·4–568·3)	4·8 (3·8–5·9)	77 700 (53 700–107 000)	14·0 (9·9–19·5)	5·1 (2·9–7·3)
**Central sub-Saharan Africa**	274 000 (216 000–346 000)	163.0 (146.8–175.2)	429·9 (334·3–535·5)	2·0 (-3·6–7·1)	8540 (5950–12 100)	13·1 (9·2–18·4)	2·6 (-6·6–12·2)
Angola	58 300 (46 200–73 200)	223.1 (201.1–243.7)	429·7 (335·3–533·0)	5·5 (0·2–11·9)	1830 (1300–2540)	13·2 (9·3–17·8)	5·0 (-9·3–19·5)
Central African Republic	10 300 (8000–12 800)	104.5 (90.7–117.7)	399·9 (314·2–494·1)	2·0 (-5·5–7·4)	322 (213–454)	12·1 (8·3–16·9)	2·5 (-11·5–19·4)
Congo (Brazzaville)	15 400 (12 100–19 900)	223.1 (198.3–249.6)	491·1 (375·3–617·8)	18·1 (11·9–24·0)	484 (320–698)	15·1 (10·4–21·9)	18·0 (3·9–35·3)
DR Congo	181 000 (142 000–228 000)	147.4 (130.9–164.2)	423·8 (328·9–528·6)	-1·1 (-7·1–6·4)	5610 (3890–7840)	12·9 (9·0–18·2)	-0·4 (-11·2–12·6)

Equatorial Guinea	3060 (2400–3800)	279.2 (247.8–311.8)	500·3 (383·0–627·3)	30·8 (22·8–40·5)	97.2 (65.9–136)	15·4 (10·6–21·5)	33·0 (16·6–54·1)
Gabon	6170 (4750–7960)	148.8 (131.4–163.0)	516·4 (398·1–647·9)	21·5 (13·6–29·0)	192 (131–270)	15·8 (10·8–21·6)	20·6 (5·4–35·3)
**Eastern sub-Saharan Africa**	866 000 (682 000–1 100 000)	149.6 (143.1–155.5)	450·3 (351·4–563·4)	6·0 (3·7–7·4)	27 300 (19 000–37 700)	13·9 (9·9–19·2)	6·6 (2·7–9·9)
Burundi	23 500 (18 600–29 700)	133.6 (116.1–150.1)	434·2 (337·2–532·8)	8·9 (1·4–16·9)	744 (534–1050)	13·4 (9·7–18·7)	9·6 (-5·0–24·5)
Comoros	2390 (1890–3060)	140.3 (126.3–154.6)	448·4 (352·1–573·5)	5·0 (-1·0–10·6)	75.4 (52.5–105)	14·0 (10·0–19·5)	5·6 (-9·0–18·1)
Djibouti	3500 (2720–4550)	377.8 (343.8–406.1)	472·1 (361·9–599·5)	13·2 (7·1–19·0)	111 (71–163)	14·6 (9·5–20·8)	13·2 (-0·4–26·2)
Eritrea	13 400 (10 700–17 100)	204.5 (188.2–225.8)	404·4 (315·9–511·2)	8·5 (2·2–15·1)	421 (276–598)	12·3 (8·5–17·0)	7·6 (-4·5–24·4)
Ethiopia	223 000 (175 000–276 000)	137.8 (126.1–148.1)	460·7 (362·8–576·4)	7·0 (2·9–11·0)	7000 (4760–9660)	14·2 (9·9–19·3)	7·9 (0·8–15·8)
Kenya	127 000 (99 900–163 000)	210.2 (202.0–217.4)	480·5 (375·9–604·1)	6·4 (4·9–7·6)	4020 (2730–5620)	14·8 (10·4–20·8)	6·6 (4·2–9·2)
Madagascar	57 000 (44 900–75 300)	145.6 (128.0–166.0)	435·0 (345·1–569·3)	2·8 (-4·3–11·4)	1810 (1200–2640)	13·5 (9·2–19·3)	2·6 (-11·8–17·6)
Malawi	37 100 (29 100–47 800)	114.6 (102.1–128.2)	431·9 (338·3–542·2)	5·4 (0·0–11·2)	1180 (760–1660)	13·4 (9·1–18·4)	7·0 (-6·5–24·0)
Mozambique	53 600 (41 500–68 300)	106.6 (92.2–122.1)	419·3 (325·9–528·1)	4·8 (-0·8–11·8)	1680 (1230–2380)	12·8 (9·1–17·5)	5·5 (-6·2–20·6)
Rwanda	29 600 (23 500–37 200)	136.6 (123.0–150.9)	416·5 (322·9–518·1)	4·7 (-0·3–9·6)	932 (652–1280)	12·8 (9·1–17·7)	3·4 (-10·2–19·0)
Somalia	31 700 (25 100–40 400)	169.0 (149.9–190.4)	383·4 (296·7–488·4)	-4·2 (-10·4–3·0)	1010 (682–1440)	11·8 (8·3–16·3)	-3·7 (-14·9–8·6)
South Sudan	21 000 (16 500–26 300)	74.1 (62.3–84.7)	478·6 (378·0–584·1)	3·7 (-2·6–10·7)	654 (452–918)	14·5 (10·4–20·3)	3·7 (-7·7–15·0)
Uganda	72 400 (57 200–92 700)	150.0 (136.2–167.1)	428·2 (339·1–530·9)	4·5 (-0·9–11·6)	2290 (1 570–3200)	13·2 (9·2–18·7)	5·4 (-9·1–19·9)
Tanzania	132 000 (106 000–165 000)	162.3 (149.0–181.1)	465·3 (355·9–575·5)	8·4 (2·7–14·5)	4190 (2940–5820)	14·5 (10·2–20·1)	9·7 (-1·0–24·8)
Zambia	38 200 (30 500–47 000)	175.9 (158.1–198.0)	464·8 (357·9–568·7)	4·8 (-0·4–12·5)	1200 (860–1670)	14·3 (10·4–20·0)	5·4 (-8·9–22·5)
**Southern sub-Saharan Africa**	342 000 (265 000–436 000)	129.7 (124.8–134.5)	549·5 (425·1–684·6)	9·1 (6·9–11·0)	10 600 (7250–14 700)	16·8 (11·7–23·4)	8·0 (3·0–12·7)

Botswana	8610<br>(6680–11 300)	219.2<br>(196.7–242.4)	522·1<br>(407·4–680·2)	20·7<br>(13·2–28·8)	269<br>(177–374)	16·0<br>(10·7–22·0)	19·0<br>(4·5–33·5)
Eswatini	3560<br>(2760–4600)	117.1<br>(105.5–131.2)	530·5<br>(408·4–666·7)	7·6<br>(1·4–14·9)	110<br>(75.8–151)	16·1<br>(11·3–22·1)	5·9<br>(-4·3–20·4)
Lesotho	6820<br>(5280–8610)	51.9<br>(41.7–62.8)	481·5<br>(375·0–593·2)	11·6<br>(5·2–19·0)	210<br>(148–296)	14·5<br>(10·4–20·0)	9·0<br>(-3·9–24·1)
Namibia	7720<br>(5960–9830)	124.6<br>(110.4–139.3)	488·4<br>(372·2–616·6)	7·9<br>(1·3–14·8)	241<br>(161–337)	15·0<br>(10·1–20·5)	7·8<br>(-7·2–22·7)
South Africa	279 000<br>(215 000–354 000)	140.7<br>(134.7–148.1)	570·4<br>(441·2–710·3)	10·0<br>(7·7–12·4)	8620<br>(5900–12 000)	17·4<br>(12·2–24·3)	9·0<br>(3·6–14·6)
Zimbabwe	36 300<br>(29 000–45 600)	75.3<br>(64.6–88.4)	449·5<br>(355·4–551·7)	-2·4<br>(-8·1–3·4)	1130<br>(797–1580)	13·7<br>(9·5–18·5)	-3·6<br>(-16·9–8·9)
**Western sub-Saharan Africa**	986 000<br>(775 000–1 250 000)	141.8<br>(136.3–147.4)	440·7<br>(341·9–550·4)	3·5<br>(1·7–5·0)	31 200<br>(21 300–43 500)	13·6<br>(9·5–19·1)	3·9<br>(1·1–6·4)
Benin	26 800<br>(21 100–34 200)	206.5<br>(184.5–229.4)	459·7<br>(356·8–576·9)	13·0<br>(5·5–19·2)	848<br>(585–1210)	14·2<br>(10·1–20·1)	13·7<br>(-0·4–27·2)
Burkina Faso	44 600<br>(35 200–57 100)	152.9<br>(133.8–170.5)	414·8<br>(323·8–528·1)	8·2<br>(0·6–15·3)	1410<br>(965–2060)	12·9<br>(9·1–18·3)	9·6<br>(-3·9–26·4)
Cabo Verde	2220<br>(1760–2770)	159.5<br>(138.4–185.0)	466·6<br>(364·0–580·6)	22·2<br>(15·2–30·1)	69.2<br>(47.1–96.7)	14·4<br>(10·0–20·3)	20·3<br>(1·3–41·9)
Cameroon	72 600<br>(57 000–92 000)	225.7<br>(204.2–244.4)	503·5<br>(390·0–626·6)	10·6<br>(3·6–17·6)	2290<br>(1530–3350)	15·6<br>(10·6–22·6)	11·6<br>(-4·4–27·4)
Chad	27 900<br>(22 000–34 300)	146.7<br>(128.6–164.4)	428·3<br>(331·5–526·3)	12·2<br>(5·5–21·2)	873<br>(602–1220)	13·1<br>(9·0–18·6)	11·3<br>(-5·0–28·5)
Côte d'Ivoire	62 200<br>(48 500–77 000)	193.9<br>(173.3–214.9)	471·4<br>(360·4–573·3)	6·9<br>(-0·6–13·4)	1960<br>(1330–2780)	14·5<br>(10·1–19·9)	7·7<br>(-5·3–20·6)
The Gambia	5070<br>(4000–6440)	195.3<br>(175.6–218.2)	454·8<br>(352·8–575·7)	7·6<br>(0·6–16·5)	160<br>(111–227)	14·1<br>(10·0–20·1)	6·9<br>(-7·3–22·3)
Ghana	88 200<br>(68 300–112 000)	214.9<br>(191.5–234.0)	463·9<br>(356·1–578·1)	16·6<br>(8·4–23·8)	2790<br>(1950–3980)	14·4<br>(10·2–20·2)	16·7<br>(0·4–30·3)
Guinea	26 600<br>(20 700–34 300)	89.8<br>(78.8–100.6)	433·2<br>(335·1–553·7)	6·6<br>(-0·5–14·0)	844<br>(575–1210)	13·5<br>(9·5–19·1)	7·5<br>(-2·1–19·4)
Guinea-Bissau	3570<br>(2840–4610)	105.7<br>(92.7–118.3)	408·1<br>(326·0–508·2)	3·9<br>(-2·7–9·6)	112<br>(75.8–155)	12·5<br>(8·5–17·4)	3·7<br>(-9·1–17·9)
Liberia	12 400<br>(9740–15 900)	139.4<br>(118.5–160.7)	488·9<br>(376·0–620·3)	8·8<br>(2·2–15·6)	385<br>(258–548)	14·9<br>(10·4–20·4)	8·2<br>(-2·9–19·4)
Mali	43 200<br>(34 200–53 700)	149.4<br>(134.1–167.9)	440·9<br>(344·0–553·3)	12·1<br>(6·0–20·3)	1360<br>(929–1920)	13·6<br>(9·5–19·0)	12·8<br>(-1·6–26·2)

Mauritania	11 700<br>(9150–14 400)	153.3<br>(135.3–168.5)	503·6<br>(390·2–614·6)	16·9<br>(9·0–23·7)	368<br>(259–495)	15·7<br>(11·3–21·0)	17·1<br>(3·6–33·3)
Niger	37 400<br>(29 800–46 600)	188.7<br>(174.2–205.4)	412·5<br>(324·7–504·6)	1·9<br>(-3·8–8·1)	1200<br>(872–1680)	12·9<br>(9·3–17·7)	3·0<br>(-9·2–15·8)
Nigeria	447 000<br>(352 000–564 000)	111.2<br>(104.0–118.6)	427·5<br>(333·3–536·8)	-3·1<br>(-4·5–-1·2)	14 200<br>(9540–19 500)	13·2<br>(9·2–18·3)	-2·7<br>(-5·7–0·3)
São Tomé and Príncipe	610<br>(483–785)	120.9<br>(104.3–140.4)	489·6<br>(375·2–619·2)	17·0<br>(10·6–23·3)	19.5<br>(12.9–27.5)	15·3<br>(10·5–21·6)	17·1<br>(0·5–33·2)
Senegal	37 500<br>(29 300–46 300)	156.2<br>(139.1–173.9)	443·1<br>(345·2–541·2)	5·6<br>(-1·7–12·7)	1190<br>(849–1700)	13·8<br>(9·9–19·4)	6·6<br>(-6·3–22·0)
Sierra Leone	18 100<br>(14 500–23 000)	122.0<br>(106.4–137.1)	430·7<br>(335·6–535·6)	6·5<br>(0·6–13·5)	574<br>(400–808)	13·4<br>(9·4–18·6)	6·9<br>(-6·6–23·3)
Togo	18 500<br>(14 700–23 600)	228.6<br>(210.1–252.4)	414·4<br>(327·3–523·9)	5·0<br>(-0·3–12·5)	587<br>(400–821)	12·9<br>(9·2–17·4)	5·1<br>(-5·6–17·0)

Note: Super-region (capitalised) and region (bold) numbers do not sum to the global prevalence due to rounding and modelling adjustments for nations with populations below 50,000

UI = Uncertainty Interval

YLD=Years lived with disability

Supplemental Table S8: Gout years lived with disability percentage attributable to risk factors by region and sex

	YLDs			YLDs		
	High body-mass index*			Kidney dysfunction		
**Location**	**Male**	**Female**	**Both**	**Male**	**Female**	**Both**
Central Asia	38·7 (31·1–47·0)	45·4 (36·9–53·8)	40·9 (32·9–49·2)	16·9 (13·4–19·8)	23·0 (18·7–27·1)	18·0 (14·3–21·1)
Central Europe	43·4 (34·7–51·8)	44·4 (36·4–52·9)	43·8 (35·4–52·0)	11·5 (8·9–14·0)	14·0 (10·9–16·8)	11·7 (9·0–14·1)
Eastern Europe	41·7 (32·8–49·2)	47·9 (39·4–56·6)	43·7 (34·8–51·3)	11·5 (8·7–13·9)	15·3 (11·8–18·2)	12·0 (9·2–14·5)
Australasia	46·3 (37·2–54·5)	46·2 (38·0–54·1)	46·4 (37·4–54·4)	10·3 (8·1–12·9)	20·8 (16·2–25·2)	12·3 (9·7–15·1)
High-income Asia Pacific	27·6 (22·1–32·9)	27·0 (21·6–31·9)	27·6 (22·2–32·8)	13·2 (10·3–16·0)	16·7 (13·0–20·2)	13·4 (10·3–16·2)
High-income North America	46·8 (37·8–55·3)	45·6 (37·3–53·4)	46·6 (37·7–54·9)	12·5 (10·2–15·0)	22·8 (18·9–26·5)	14·1 (11·5–16·7)
Southern Latin America	45·4 (36·1–54·3)	47·9 (39·0–56·6)	46·0 (36·8–54·8)	8·0 (6·0–10·1)	13·1 (10·2–16·3)	8·7 (6·6–10·9)
Western Europe	41·3 (33·1–50·0)	41·5 (33·2–49·5)	41·4 (33·1–49·9)	10·4 (8·0–13·0)	17·0 (13·4–20·2)	11·3 (8·9–13·9)
Andean Latin America	41·2 (33·2–49·8)	44·7 (36·6–52·5)	42·2 (34·3–50·7)	8·0 (6·2–9·7)	9·2 (7·1–11·7)	8·3 (6·4–10·2)
Caribbean	37·2 (29·9–44·8)	42·7 (34·7–50·6)	39·2 (31·6–46·7)	10·6 (8·1–13·1)	8·8 (6·6–10·8)	9·8 (7·4–12·2)
Central Latin America	44·2 (35·4–52·6)	47·1 (38·7–55·6)	45·3 (36·7–53·6)	12·2 (9·4–14·9)	13·1 (10·1–15·9)	12·5 (9·6–15·2)
Tropical Latin America	41·3 (32·7–49·0)	43·1 (34·8–50·9)	42·0 (33·5–49·8)	15·9 (12·8–19·1)	11·3 (8·8–13·8)	13·9 (11·1–16·8)
North Africa and Middle East	44·8 (36·4–52·8)	50·3 (41·7–59·0)	46·2 (37·8–54·3)	11·5 (9·0–14·0)	13·8 (10·8–16·7)	12·0 (9·4–14·6)
South Asia	18·9 (15·9–22·9)	23·6 (19·0–28·4)	20·2 (16·7–24·3)	11·9 (9·2–14·3)	13·5 (10·6–16·3)	12·2 (9·5–14·6)
East Asia	29·7 (23·5–35·6)	33·1 (26·6–39·6)	30·6 (24·3–36·6)	7·7 (5·8–9·5)	9·5 (7·2–11·5)	7·9 (6·0–9·7)
Oceania	34·7 (27·7–41·3)	38·5 (31·6–45·5)	35·7 (28·7–42·2)	11·1 (8·7–13·5)	12·4 (9·8–14·9)	11·3 (8·9–13·6)
Southeast Asia	21·2 (17·4–25·5)	27·6 (22·6–33·3)	22·9 (18·8–27·4)	11·7 (9·2–14·0)	12·6 (9·9–15·2)	11·4 (9·0–13·7)
Central sub-Saharan Africa	26·4 (21·1–31·7)	29·4 (23·7–35·6)	27·3 (21·9–32·5)	20·7 (16·5–24·6)	16·9 (13·4–20·0)	18·8 (15·1–22·4)
Eastern sub-Saharan Africa	20·8 (17·1–25·0)	26·6 (21·6–32·2)	22·5 (18·4–27·0)	7·0 (5·4–8·6)	7·5 (5·7–9·2)	7·0 (5·4–8·6)
Southern sub-Saharan Africa	38·1 (30·6–45·7)	48·9 (40·2–57·8)	41·1 (33·2–48·9)	18·0 (14·5–21·6)	18·0 (14·2–21·4)	17·2 (13·8–20·5)
Western sub-Saharan Africa	29·3 (23·7–34·5)	34·8 (28·0–41·6)	30·8 (24·8–36·4)	15·7 (12·5–18·6)	14·9 (11·8–17·9)	15·4 (12·3–18·3)

* High body mass index defined as ≥25 kg/m^2

Supplemental Table S9: Forecast of gout age-standardised prevalence per 100,000 and total cases globally and by region, both sexes, 2020–2050

	Age-standardised prevalence (per 100,000)			Cases (millions)		
**Region**	**2030**	**2040**	**2050**	**2030**	**2040**	**2050**
Global	665 (530–828)	665 (531–828)	667 (531–830)	70·1 (57·9–85·5)	84·0 (70·5–101)	95·8 (81·1–116)
Andean Latin America	298 (238–374)	307 (248–382)	316 (257–391)	0·235 (0·196–0·288)	0·305 (0·257–0·366)	0·376 (0·306–0·454)
Australasia	1440 (1150–1870)	1470 (1180–1900)	1500 (1210–1940)	0·812 (0·649–1·03)	0·949 (0·770–1·22)	1·07 (0·851–1·41)
Caribbean	251 (204–306)	257 (210–311)	263 (216–317)	0·156 (0·129–0·185)	0·183 (0·154–0·220)	0·207 (0·172–0·259)
Central Asia	450 (348–559)	463 (363–571)	476 (378–584)	0·474 (0·388–0·580)	0·588 (0·482–0·704)	0·698 (0·562–0·845)
Central Europe	376 (299–472)	386 (308–482)	394 (316–490)	0·772 (0·626–0·951)	0·807 (0·669–0·973)	0·812 (0·675–0·988)
Central Latin America	194 (155–236)	200 (162–242)	206 (169–248)	0·620 (0·522–0·742)	0·772 (0·660–0·927)	0·912 (0·760–1·09)
Central sub-Saharan Africa	461 (363–564)	488 (390–589)	510 (412–614)	0·439 (0·361–0·533)	0·693 (0·564–0·881)	1·05 (0·806–1·37)
East Asia	852 (677–1060)	887 (712–1100)	928 (753–1140)	21·7 (17·6–26·5)	24·8 (20·8–29·9)	25·9 (21·6–31·8)
Eastern Europe	445 (348–557)	460 (365–570)	473 (378–582)	1·56 (1·28–1·91)	1·69 (1·39–2·04)	1·76 (1·41–2·16)
Eastern sub-Saharan Africa	475 (378–590)	501 (401–616)	526 (425–642)	1·38 (1·16–1·67)	2·20 (1·82–2·74)	3·41 (2·65–4·26)
High-income Asia Pacific	748 (598–954)	764 (618–971)	779 (636–985)	3·02 (2·40–3·72)	3·18 (2·58–3·81)	3·16 (2·60–3·87)
High-income North America	1740 (1470–2100)	1760 (1490–2120)	1780 (1520–2150)	11·2 (9·61–13·3)	12·2 (10·5–14·5)	12·8 (11·0–15·3)
North Africa and Middle East	548 (434–680)	570 (456–703)	588 (473–720)	3·79 (3·16–4·62)	5·28 (4·36–6·39)	6·85 (5·55–8·30)
Oceania	711 (564–897)	729 (581–915)	754 (605–940)	0·0831 (0·0685–0·105)	0·114 (0·0949–0·139)	0·154 (0·126–0·183)
South Asia	443 (351–554)	465 (373–577)	484 (391–597)	8·78 (7·27–10·7)	11·8 (9·97–14·5)	15·0 (12·6–18·3)
Southeast Asia	667 (528–842)	693 (553–867)	718 (578–891)	5·82 (4·84–7·19)	7·31 (6·14–8·86)	8·58 (7·11–10·5)
Southern Latin America	950 (766–1220)	978 (794–1250)	1000 (821–1270)	0·933 (0·776–1·16)	1·12 (0·942–1·35)	1·28 (1·06–1·54)
Southern sub-Saharan Africa	571 (448–707)	592 (467–728)	609 (484–746)	0·468 (0·385–0·566)	0·624 (0·519–0·739)	0·799 (0·647–0·957)
Tropical Latin America	264 (213–325)	273 (223–334)	282 (232–343)	0·839 (0·698–1·01)	1·03 (0·868–1·26)	1·20 (0·992–1·44)
Western Europe	640 (514–816)	653 (528–829)	666 (542–842)	5·56 (4·41–6·89)	6·06 (4·89–7·40)	6·29 (5·02–7·66)
Western sub-Saharan Africa	460 (360–571)	483 (383–594)	508 (406–619)	1·51 (1·27–1·83)	2·35 (1·95–2·87)	3·57 (2·86–4·45)

Input data sources

National Institute of Public Health (Mexico). Mexico National Health Survey 1999-2000.

Australian Bureau of Statistics. Australia National Health Survey 1995. Canberra, Australia: Australian Bureau of Statistics.

National Center for Health Statistics (NCHS), Centers for Disease Control and Prevention (CDC), US Census Bureau. United States National Health Interview Survey 1969. Hyattsville, United States: National Center for Health Statistics (NCHS), Centers for Disease Control and Prevention (CDC).

National Center for Health Statistics (NCHS), Centers for Disease Control and Prevention (CDC), US Census Bureau. United States National Health Interview Survey 1988. Hyattsville, United States: National Center for Health Statistics (NCHS), Centers for Disease Control and Prevention (CDC).

National Center for Health Statistics (NCHS), Centers for Disease Control and Prevention (CDC), US Census Bureau. United States National Health Interview Survey 1992. Hyattsville, United States: National Center for Health Statistics (NCHS), Centers for Disease Control and Prevention (CDC).

National Center for Health Statistics (NCHS), Centers for Disease Control and Prevention (CDC), US Census Bureau. United States National Health Interview Survey 1996. Hyattsville, United States: National Center for Health Statistics (NCHS), Centers for Disease Control and Prevention (CDC).

National Center for Health Statistics (NCHS), Centers for Disease Control and Prevention (CDC). United States National Health and Nutrition Examination Survey 2009-2010. Hyattsville, United States: National Center for Health Statistics (NCHS), Centers for Disease Control and Prevention (CDC), 2011.

Chaiamnuay P, Darmawan J, Muirden KD, Assawatanabodee P. Epidemiology of rheumatic disease in rural Thailand: a WHO-ILAR COPCORD study. Community Oriented Programme for the Control of Rheumatic Disease. J Rheumatol. 1998; 25(7): 1382-7.

Davatchi F, Jamshidi A-R, Banihashemi AT, Gholami J, Forouzanfar MH, Akhlaghi M, Barghamdi M, Noorolahzadeh E, Khabazi A-R, Salesi M, Salari A-H, Karimifar M, Essalat-Manesh K, Hajialiloo M, Soroosh M, Farzad F, Moussavi H-R, Samadi F, Ghaznavi K, Asgharifard H, Zangiabadi A-H, Shahram F, Nadji A, Akbarian M, Gharibdoost F. WHO-ILAR COPCORD Study (Stage 1, Urban Study) in Iran. J Rheumatol. 2008; 35(7): 1384.

De Vera M, Rahman MM, Rankin J, Kopec J, Gao X, Choi H. Gout and the risk of Parkinson's disease: a cohort study. Arthritis Rheum. 2008; 59(11): 1549-54.

Mikuls TR, Farrar JT, Bilker WB, Fernandes S, Schumacher HR Jr, Saag KG. Gout epidemiology: results from the UK General Practice Research Database, 1990-1999. Ann Rheum Dis. 2005; 64(2): 267-72.

Arromdee E, Michet CJ, Crowson CS, O'Fallon WM, Gabriel SE. Epidemiology of gout: is the incidence rising? J Rheumatol. 2002; 29(11): 2403-6.

Hanova P, Pavelka K, Dostal C, Holcatova I, Pikhart H. Epidemiology of rheumatoid arthritis, juvenile idiopathic arthritis and gout in two regions of the Czech Republic in a descriptive population-based survey in 2002-2003. Clin Exp Rheumatol. 2006; 24(5): 499-507.

Choi HK, Atkinson K, Karlson EW, Willett W, Curhan G. Purine-rich foods, dairy and protein intake, and the risk of gout in men. N Engl J Med. 2004; 350(11): 1093-103.

Currie WJ. Prevalence and incidence of the diagnosis of gout in Great Britain. Ann Rheum Dis. 1979; 38(2): 101-6.

Cea Soriano L, Rothenbacher D, Choi HK, García Rodríguez LA. Contemporary epidemiology of gout in the UK general population. Arthritis Res Ther. 2011; 13(2): R39.

Roubenoff R, Klag MJ, Mead LA, Liang KY, Seidler AJ, Hochberg MC. Incidence and risk factors for gout in white men. JAMA. 1991; 266(21): 3004-7.

Al-Awadhi AM, Olusi SO, Moussa M, Shehab D, Al-Zaid N, Al-Herz A, Al-Jarallah K. Musculoskeletal pain, disability and health-seeking behavior in adult Kuwaitis using a validated Arabic version of the WHO-ILAR COPCORD Core Questionnaire. Clin Exp Rheumatol. 2004; 22(2): 177-83.

Andrianakos A, Trontzas P, Christoyannis F, Dantis P, Voudouris C, Georgountzos A, Kaziolas G, Vafiadou E, Pantelidou K, Karamitsos D, Kontelis L, Krachtis P, Nikolia Z, Kaskani E, Tavaniotou E, Antoniades C, Karanikolas G, Kontoyanni A, ESORDIG Study. Prevalence of rheumatic diseases in Greece: a cross-sectional population based epidemiological study. The ESORDIG Study. J Rheumatol. 2003; 30(7): 1589-601.

Cardiel MH, Rojas-Serrano J. Community based study to estimate prevalence, burden of illness and help seeking behavior in rheumatic diseases in Mexico City. A COPCORD study. Clin Exp Rheumatol. 2002; 20(5): 617-24.

Haq SA, Darmawan J, Islam MN, Uddin MZ, Das BB, Rahman F, Chowdhury MAJ, Alam MN, Mahmud TAK, Chowdhury MR, Tahir M. Prevalence of rheumatic diseases and associated outcomes in rural and urban communities in Bangladesh: a COPCORD study. J Rheumatol. 2005; 32(2): 348-53.

Lin KC, Lin HY, Chou P. Community based epidemiological study on hyperuricemia and gout in Kin-Hu, Kinmen. J Rheumatol. 2000; 27(4): 1045-50.

Zeng Q, Huang S, Chen R. 10-year epidemiological study on rheumatic diseases in Shantou area. Chin J Intern Med. 1997; 36(3): 193-7.

Jackson L, Taylor R, Faaiuso S, Ainuu SP, Whitehouse S, Zimmet P. Hyperuricaemia and gout in Western Samoans. J Chronic Dis. 1981; 34(2-3): 65-75.

Bergström G, Bjelle A, Sorensen LB, Sundh V, Svanborg A. Prevalence of rheumatoid arthritis, osteoarthritis, chondrocalcinosis and gouty arthritis at age 79. J Rheumatol. 1986; 13(3): 527-34.

Chou CT, Pei L, Chang DM, Lee CF, Schumacher HR, Liang MH. Prevalence of rheumatic diseases in Taiwan: a population study of urban, suburban, rural differences. J Rheumatol. 1994; 21(2): 302-6.

Dai S-M, Han X-H, Zhao D-B, Shi Y-Q, Liu Y, Meng J-M. Prevalence of rheumatic symptoms, rheumatoid arthritis, ankylosing spondylitis, and gout in Shanghai, China: a COPCORD study. J Rheumatol. 2003; 30(10): 2245-51.

Alvarez Nemegyei J, Nuño Gutiérrez BL, Alcocer Sánchez JA. Rheumatic diseases and labor disability in adult rural population. Rev Med Inst Mex Seguro Soc. 2005; 43(4): 287-92.

González Buitrago JM, Arroyo A, Vega L, García Fernández N, De la Rua A, Navajo JA. Prevalence of excess blood uric acid and gout in a sample of a rural Castillian population 40-50 years of age. Rev Clin Esp. 1988; 182(9): 454-8.

Chen S, Du H, Wang Y, Xu L. The epidemiology study of hyperuricemia and gout in a community population of Huangpu District in Shanghai. Chin Med J (Engl). 1998; 111(3): 228-30.

Tsitlanadze VG, Kartvelishvili EI, Shakulashvili NA, Shalamberidze LP. Incidence and various risk factors for gout in the Georgian SSR. Ter Arkh. 1987; 59(4): 18-20.

Darmawan J, Valkenburg HA, Muirden KD, Wigley RD. The epidemiology of gout and hyperuricemia in a rural population of Java. J Rheumatol. 1992; 19(10): 1595-9.

Farooqi A, Gibson T. Prevalence of the major rheumatic disorders in the adult population of north Pakistan. Br J Rheumatol. 1998; 37(5): 491-5.

Reyes-Llerena GA, Guibert-Toledano M, Penedo-Coello A, Pérez-Rodríguez A, Baez-Dueñas RM, Charnicharo-Vidal R, Cardiel MH. Community-based study to estimate prevalence and burden of illness of rheumatic diseases in Cuba: a COPCORD study. J Clin Rheumatol. 2009; 15(2): 51-5.

Steven MM. Prevalence of chronic arthritis in four geographical areas of the Scottish Highlands. Ann Rheum Dis. 1992; 51(2): 186-94.

Wigley R, Manahan L, Muirden KD, Caragay R, Pinfold B, Couchman KG, Valkenburg HA. Rheumatic disease in a Philippine village. II: a WHO-ILAR-APLAR COPCORD study, phases II and III. Rheumatol Int. 1991; 11(4-5): 157-61.

Annemans L, Spaepen E, Gaskin M, Bonnemaire M, Malier V, Gilbert T, Nuki G. Gout in the UK and Germany: prevalence, comorbidities and management in general practice 2000-2005. Ann Rheum Dis. 2008; 67(7): 960-6.

Novak S, Melkonian AK, Patel PA, Kleinman NL, Joseph-Ridge N, Brook RA. Metabolic syndrome-related conditions among people with and without gout: prevalence and resource use. Curr Med Res Opin. 2007; 23(3): 623-30.

Wallace KL, Riedel AA, Joseph-Ridge N, Wortmann R. Increasing prevalence of gout and hyperuricemia over 10 years among older adults in a managed care population. J Rheumatol. 2004; 31(8): 1582-7.

Deesomchok U, Tumrasvin T. Common arthritis in practice. J Med Assoc Thai. 1988; 71(12): 671-6.

Klemp P, Stansfield SA, Castle B, Robertson MC. Gout is on the increase in New Zealand. Ann Rheum Dis. 1997; 56(1): 22-6.

Miao Z, Li C, Chen Y, Zhao S, Wang Y, Wang Z, Chen X, Xu F, Wang F, Sun R, Hu J, Song W, Yan S, Wang C-Y. Dietary and lifestyle changes associated with high prevalence of hyperuricemia and gout in the Shandong coastal cities of Eastern China. J Rheumatol. 2008; 35(9): 1859-64.

Minh Hoa TT, Darmawan J, Chen SL, Van Hung N, Thi Nhi C, Ngoc An T, Damarwan J, Shun Le C. Prevalence of the rheumatic diseases in urban Vietnam: a WHO-ILAR COPCORD study. J Rheumatol. 2003; 30(10): 2252-6.

Salaffi F, De Angelis R, Grassi W, MArche Pain Prevalence, INvestigation Group (MAPPING) study. Prevalence of musculoskeletal conditions in an Italian population sample: results of a regional community-based study. I. The MAPPING study. Clin Exp Rheumatol. 2005; 23(6): 819-28.

Shi F, Gu K, Lu W, Weng W, Zhu M, Peng Y, Fu D, Fu H. Study on the prevalence of arthritis and relevant factors in Shanghai. Chin J Epidemiol. 2003; 24(12): 1136-40.

Wang Q, Chen R, Du L, Zeng Q. An epidemiological and clinical study of primary gout. Chin J Intern Med. 2001; 40(5): 313-5.

Zhang QH, Jiang ZX, Sun Y, Xia AX, Lin H, Tian L. Investigation and analysis of disease prevalence among 20 500 urban middle-aged and elderly women in five communities of Beijing. Chin J Clin Rehab. 2005; 9(39): 20-22.

Harris CM, Lloyd DC, Lewis J. The prevalence and prophylaxis of gout in England. J Clin Epidemiol. 1995; 48(9): 1153-8.

Picavet HSJ, Hazes JMW. Prevalence of self-reported musculoskeletal diseases is high. Ann Rheum Dis. 2003; 62(7): 644-50.

Collins JG, National Center for Health Statistics (NCHS), Centers for Disease Control and Prevention (CDC). Prevalence of selected chronic conditions, United States, 1983-85. Hyattsville, MD: National Center for Health Statistics (NCHS), Centers for Disease Control and Prevention (CDC); 1988. 16 p. (Advance data from vital and health statistics; No. 155. DHHS Pub. No. (PHS) 88-1250. Public Health Service).

Zhu Y, Pandya BJ, Choi HK. Prevalence of gout and hyperuricemia in the US general population: the National Health and Nutrition Examination Survey 2007-2008. Arthritis Rheum. 2011; 63(10): 3136-41.

Chang HY, Pan WH, Yeh WT, Tsai KS. Hyperuricemia and gout in Taiwan: results from the Nutritional and Health Survey in Taiwan (1993-96). J Rheumatol. 2001; 28(7): 1640-6.

Gardner MJ, Power C, Barker DJ, Padday R. The prevalence of gout in three English towns. Int J Epidemiol. 1982; 11(1): 71-5.

De Vera MA, Rahman MM, Bhole V, Kopec JA, Choi HK. Independent impact of gout on the risk of acute myocardial infarction among elderly women: a population-based study. Ann Rheum Dis. 2010; 69(6): 1162-4.

Choi HK, Curhan G. Independent impact of gout on mortality and risk for coronary heart disease. Circulation. 2007; 116(8): 894-900.

Krishnan E, Svendsen K, Neaton JD, Grandits G, Kuller LH, MRFIT Research Group. Long-term cardiovascular mortality among middle-aged men with gout. Arch Intern Med. 2008; 168(10): 1104-10.

Dans LF, Tankeh-Torres S, Amante CM, Penserga EG. The prevalence of rheumatic diseases in a Filipino urban population: a WHO-ILAR COPCORD Study. World Health Organization. International League of Associations for Rheumatology. Community Oriented Programme for the Control of the Rheumatic Diseases. J Rheumatol. 1997; 24(9): 1814-9.

Ministry of Health (New Zealand), National Research Bureau Ltd (New Zealand). New Zealand Health Survey 2006-2007.

Anagnostopoulos I, Zinzaras E, Alexiou I, Papathanasiou AA, Davas E, Koutroumpas A, Barouta G, Sakkas LI. The prevalence of rheumatic diseases in central Greece: a population survey. BMC Musculoskelet Disord. 2010; 98.

Li R, Sun J, Ren L-M, Wang H-Y, Liu W-H, Zhang X-W, Chen S, Mu R, He J, Zhao Y, Long L, Liu Y-Y, Liu X, Lu X-L, Li Y-H, Wang S-Y, Pan S-S, Li C, Wang H-Y, Li Z-G. Epidemiology of eight common rheumatic diseases in China: a large-scale cross-sectional survey in Beijing. Rheumatology (Oxford). 2012; 51(4): 721-9.

Bhole V, de Vera M, Rahman MM, Krishnan E, Choi H. Epidemiology of gout in women: Fifty-two-year followup of a prospective cohort. Arthritis Rheum. 2010; 62(4): 1069-76.

Chen J-H, Yeh W-T, Chuang S-Y, Wu Y-Y, Pan W-H. Gender-specific risk factors for incident gout: a prospective cohort study. Clin Rheumatol. 2012; 31(2): 239-45.

Jackson G, Wright C, Thornley S, Taylor WJ, Te Karu L, Gow PJ, Arroll B, Gribben B, Dalbeth N, Winnard D. Potential unmet need for gout diagnosis and treatment: capture-recapture analysis of a national administrative dataset. Rheumatology (Oxford). 2012; 51(10): 1820-4.

Chuang S-Y, Lee S-C, Hsieh Y-T, Pan W-H. Trends in hyperuricemia and gout prevalence: Nutrition and Health Survey in Taiwan from 1993-1996 to 2005-2008. Asia Pac J Clin Nutr. 2011; 20(2): 301-8.

Rodriguez-Amado J, Peláez-Ballestas I, Sanin LH, Esquivel-Valerio JA, Burgos-Vargas R, Pérez-Barbosa L, Riega-Torres J, Garza-Elizondo MA. Epidemiology of rheumatic diseases. A community-based study in urban and rural populations in the state of Nuevo Leon, Mexico. J Rheumatol Suppl. 2011; 9-14.

Peláez-Ballestas I, Sanin LH, Moreno-Montoya J, Alvarez-Nemegyei J, Burgos-Vargas R, Garza-Elizondo M, Rodríguez-Amado J, Goycochea-Robles M-V, Madariaga M, Zamudio J, Santana N, Cardiel MH, Grupo de Estudio Epidemiológico de Enfermedades Músculo Articulares (GEEMA). Epidemiology of the rheumatic diseases in Mexico. A study of 5 regions based on the COPCORD methodology. J Rheumatol Suppl. 2011; 86: 3-8.

Statistics Norway. Norway Survey of Living Conditions 2008-2009. Oslo, Norway: Statistics Norway.

Statistics Norway. Norway Survey of Living Conditions 2005-2006. Oslo, Norway: Statistics Norway.

Alvarez-Nemegyei J, Pelaez-Ballestas I, Sanin LH, Cardiel MH, Ramirez-Angulo A, Goycochea-Robles M-V. Prevalence of musculoskeletal pain and rheumatic diseases in the southeastern region of Mexico. A COPCORD-based community survey. J Rheumatol. 2011; 86(Suppl): 21-5.

Cakır N, Pamuk ÖN, Derviş E, Imeryüz N, Uslu H, Benian Ö, Elelçi E, Erdem G, Sarvan FO, Senocak M. The prevalences of some rheumatic diseases in western Turkey: Havsa study. Rheumatol Int. 2012; 32(4): 895-908.

Chaaya M, Slim ZN, Habib RR, Arayssi T, Dana R, Hamdan O, Assi M, Issa Z, Uthman I. High burden of rheumatic diseases in Lebanon: a COPCORD study. Int J Rheum Dis. 2012; 15(2): 136-43.

Statistics Norway. Norway Survey of Living Conditions 2012-2013.

CBG Health Research Ltd., Ministry of Health (New Zealand), University of Wollongong. New Zealand Health Survey 2011-2012. Wellington, New Zealand: Ministry of Health (New Zealand).

Kuo C-F, Grainge MJ, Mallen C, Zhang W, Doherty M. Rising burden of gout in the UK but continuing suboptimal management: a nationwide population study. Ann Rheum Dis. 2014.

Lu X, Li X, Zhao Y, Zheng Z, Guan S, Chan P. Contemporary epidemiology of gout and hyperuricemia in community elderly in Beijing. Int J Rheum Dis. 2013.

Kuo C-F, Grainge MJ, See L-C, Yu K-H, Luo S-F, Valdes AM, Zhang W, Doherty M. Familial aggregation of gout and relative genetic and environmental contributions: a nationwide population study in Taiwan. Ann Rheum Dis. 2013.

Maynard JW, McAdams DeMarco MA, Baer AN, Köttgen A, Folsom AR, Coresh J, Gelber AC. Incident gout in women and association with obesity in the Atherosclerosis Risk in Communities (ARIC) Study. Am J Med. 2012; 125(7): 717.

Yu K-H, Kuo C-F, Luo S-F, See L-C, Chou I-J, Chang H-C, Chiou M-J. Risk of end-stage renal disease associated with gout: a nationwide population study. Arthritis Res Ther. 2012; 14(2): R83.

Teng GG, Ang L-W, Saag KG, Yu MC, Yuan J-M, Koh W-P. Mortality due to coronary heart disease and kidney disease among middle-aged and elderly men and women with gout in the Singapore Chinese Health Study. Ann Rheum Dis. 2012; 71(6): 924-8.

Kuo C-F, Yu K-H, See L-C, Chou I-J, Tseng W-Y, Chang H-C, Shen Y-M, Luo S-F. Elevated risk of mortality among gout patients: a comparison with the national population in Taiwan. Joint Bone Spine. 2011; 78(6): 577-80.

CBG Health Research Ltd., Ministry of Health (New Zealand). New Zealand Health Survey 2012-2013. Wellington, New Zealand: Ministry of Health (New Zealand).

CBG Health Research Ltd., Ministry of Health (New Zealand). New Zealand Health Survey 2013-2014. Wellington, New Zealand: Ministry of Health (New Zealand).

CBG Health Research Ltd., Ministry of Health (New Zealand). New Zealand Health Survey 2014-2015. Wellington, New Zealand: Ministry of Health (New Zealand).

Indian Council of Medical Research (ICMR), Ministry of Health and Family Welfare (India), Vardhman Mahavir Medical College and Safdarjung Hospital (India). India Survey on Musculoskeletal Conditions 2007-2010.

CBG Health Research Ltd., Ministry of Health (New Zealand). New Zealand Health Survey 2015-2016. Wellington, New Zealand: Ministry of Health (New Zealand).

Kapetanovic MC, Hameed M, Turkiewicz A, Neogi T, Saxne T, Jacobsson L, Englund M. Prevalence and incidence of gout in southern Sweden from the socioeconomic perspective. RMD Open. 2016; 2(2): e000326.

Dehlin M, Drivelegka P, Sigurdardottir V, Svärd A, Jacobsson LT. Incidence and prevalence of gout in Western Sweden. Arthritis Res Ther. 2016; 18: 164.

Wändell P, Carlsson AC, Ljunggren G. Gout and its comorbidities in the total population of Stockholm. Prev Med. 2015; 81: 387-91.

Guevara-Pacheco S, Feicán-Alvarado A, Sanín LH, Vintimilla-Ugalde J, Vintimilla-Moscoso F, Delgado-Pauta J, Lliguisaca-Segarra A, Dután-Erráez H, Guevara-Mosquera D, Ochoa-Robles V, Cardiel MH, Peláez-Ballestas I. Prevalence of musculoskeletal disorders and rheumatic diseases in Cuenca, Ecuador: a WHO-ILAR COPCORD study. Rheumatol Int. 2016; 36(9): 1195-1204.

Courage UU, Stephen DP, Lucius IC, Ani C, Oche AO, Emmanuel AI, Olufemi AO. Prevalence of musculoskeletal diseases in a semi-urban Nigerian community: results of a cross-sectional survey using COPCORD methodology. Clin Rheumatol. 2017; 36(11): 2509-2516.

Branco JC, Rodrigues AM, Gouveia N, Eusébio M, Ramiro S, Machado PM, da Costa LP, Mourão AF, Silva I, Laires P, Sepriano A, Araújo F, Gonçalves S, Coelho PS, Tavares V, Cerol J, Mendes JM, Carmona L, Canhão H, EpiReumaPt study group. Prevalence of rheumatic and musculoskeletal diseases and their impact on health-related quality of life, physical function and mental health in Portugal: results from EpiReumaPt- a national health survey. RMD Open. 2016; 2(1): e000166.

Zeng S-Y, Gong Y, Zhang Y-P, Chen S-B, Chen J-Y, Lin C-Q, Peng J-H, Hou Z-D, Zhong J-Q, Liang H-J, Huang G-H, Wang D-M, Lai H-Y, Li L-P, Zeng QY. Changes in the Prevalence of Rheumatic Diseases in Shantou, China, in the Past Three Decades: A COPCORD Study. PLoS One. 2015; 10(9): e0138492.

Ministry of Health (New Zealand). New Zealand Gout Prevalence 2007-2015.

Kinge JM, Knudsen AK, Skirbekk V, Vollset SE. Musculoskeletal disorders in Norway: prevalence of chronicity and use of primary and specialist health care services. BMC Musculoskelet Disord. 2015; 16(1): 75.

Weinstein, Maxine, Noreen Goldman, Ming-Cheng Chang, Hui-Sheng Lin, Yi-Li Chuang, Christine E. Peterson, Dana A. Glei, Baai-Shyun Hurng, Yu-Hsuan Lin, Shu-Hui Lin, I-Wen Liu, Hsia-Yuan Liu, Shio-Jean Lin, Chun-Ming Wu, Mei-Ling Hsiao, and Shiow-lng Wu. Social Environment and Biomarkers of Aging Study (SEBAS) in Taiwan, 2000 and 2006 /Computer file/. ICPSR 3792.vS. Ann Arbor, MI: Inter-

university Consortium/or Political and Social Research /distributor/, 2011-06-17. doi: 10.3886/ICPSR03792

Bureau of Health Promotion, Department of Health (Taiwan), Department of Health (Taiwan), Population Studies Center, University of Michigan, Union Clinical Laboratory (UCL) (Taiwan), iSTAT Healthcare Consulting (Taiwan). Taiwan Social Environment and Biomarkers of Aging Study (SEBAS) 2006-2007.

Akpabio AA, Dung-Gwom PS, Olaosebikan BH, Adelowo OO. Frequency and associations of chronic kidney disease among gout patients from a University Teaching Hospital in Nigeria. Reumatologia. 2018; 56(1): 17-23.

Ministry of Health and Welfare (Taiwan). Taiwan National Health Insurance Claims Data 2016.

National Health Fund (Poland). Poland National Health Fund Patient Claims 2015.

National Health Fund (Poland). Poland National Health Fund Patient Claims 2016.

National Health Fund (Poland). Poland National Health Fund Patient Claims 2017.

Author's contributions

Providing data or critical feedback on data sources

Aidin Abedi, Al Artaman, Maciej Banach, Akshaya Srikanth Bhagavathula, Ajay Nagesh Bhat, Peter M Brooks, Dinh-Toi Chu, Marita Cross, Xiaochen Dai, Saswati Das, Thanh Chi Do, Karsten E Dreinhoefer, Ali Fatehizadeh, Balasankar Ganesan, Mahaveer Golechha, Nima Hafezi-Nejad, Lydia M Haile, Simon I Hay, Samuel Hundessa, Shubha Jayaram, Vidya Kadashetti, Moien AB Khan, Ali Kiadaliri, Min Seo Kim, Jacek A Kopec, Kewal Krishan, Thao Thi Thu Le, Stephen S Lim, Azeem Majeed, Lyn M March, Mohamed Kamal Mesregah, Erkin M Mirrakhimov, Manish Mishra, Arup Kumar Misra, Syam Mohan, Ali H Mokdad, Mohammad Ali Moni, Yousef Moradi, Vincent Mougin, Christopher J L Murray, Sreenivas Narasimha Swamy, Van Thanh Nguyen, Robina Khan Niazi, Kanyin Liane Ong, Mayowa O Owolabi, Jagadish Rao Padubidri, Jay Patel, Shrikant Pawar, Paolo Pedersini, Salman Rawaf, Allen Seylani, Paramdeep Singh, Yonatan Solomon, Jaimie D Steinmetz, Ker-Kan Tan, Nigusie Selomon Selomon Tibebu, Theo Vos, and Anthony D Woolf.

Developing methods or computational machinery

Aleksandr Y Aravkin, Peter M Brooks, Garland T Culbreth, Xiaochen Dai, Karsten E Dreinhoefer, Lydia M Haile, Simon I Hay, Jacek A Kopec, Lyn M March, Ali H Mokdad, Vincent Mougin, Christopher J L Murray, Van Thanh Nguyen, Kanyin Liane Ong, Quinn Rafferty, Austin E Schumacher, Jaimie D Steinmetz, Theo Vos, Anthony D Woolf, and Peng Zheng.

Providing critical feedback on methods or results

Mohammadreza Abbasian, Mitra Abbasifard, Aidin Abedi, Melka Biratu Aboye, Al Artaman, Maciej Banach, Isabela M Bensenor, Neil Betteridge, Akshaya Srikanth Bhagavathula, Ajay Nagesh Bhat, Peter M Brooks, Rachelle Buchbinder, Katrin Burkart, Dinh-Toi Chu, Sheng-Chia Chung, Marita Cross, Garland T Culbreth, Omid Dadras, Xiaochen Dai, Saswati Das, Sameer Dhingra, Thanh Chi Do, Karsten E Dreinhoefer, Hisham Atan Edinur, Ali Fatehizadeh, Getahun Fetensa, Marisa Freitas, Balasankar Ganesan, Ali Gholami, Tiffany K Gill, Mahaveer Golechha, Pouya Goleij, Nima Hafezi-Nejad, Lydia M Haile, Samer Hamidi, Simon I Hay, Samuel Hundessa, Shubha Jayaram, Vidya Kadashetti, Ibraheem M Karaye, Ejaz Ahmad Khan, Moien AB Khan, Moawiah Mohammad Khatatbeh, Min Seo Kim, Ali-Asghar Kolahi, Jacek A Kopec, Kewal Krishan, Narinder Kumar, Thao Thi Thu Le, Stephen S Lim, Stany W Lobo, Azeem Majeed, Ahmad Azam Malik, Lyn M March, Mohamed Kamal Mesregah, Tomislav Mestrovic, Erkin M Mirrakhimov, Manish Mishra, Madeline E Moberg, Nouh Saad Mohamed, Syam Mohan, Ali H Mokdad, Kaveh Momenzadeh, Mohammad Ali Moni, Yousef Moradi, Christopher J L Murray, Sreenivas Narasimha Swamy, Van Thanh Nguyen, Robina Khan Niazi, Kanyin Liane Ong, Mayowa O Owolabi, Jagadish Rao Padubidri, Jay Patel, Shrikant Pawar, Paolo Pedersini, Mosiur Rahman, Mohammad-Mahdi Rashidi, Salman Rawaf, Aly M A Saad, Fatemeh Saheb Sharif-Askari, Mohamed Metwalii Khalifa Saleh, Paramdeep Singh, Amanda E Smith, Yonatan Solomon, Jaimie D Steinmetz, Ker-Kan Tan, Nigusie Selomon Selomon Tibebu, Theo Vos, Anthony D Woolf, Yuyi You, and Osama A Zitoun.

Drafting the work or revising it critically for important intellectual content

Mohammadreza Abbasian, Aidin Abedi, Maciej Banach, Isabela M Bensenor, Akshaya Srikanth Bhagavathula, Ajay Nagesh Bhat, Saeid Bitaraf, Peter M Brooks, Dinh-Toi Chu, Ewerton Cousin, Marita Cross, Garland T Culbreth, Thanh Chi Do, Karsten E Dreinhoefer, Ali Fatehizadeh, Getahun Fetensa, Marisa Freitas, Balasankar Ganesan, Tiffany K Gill, Nima Hafezi-Nejad, Lydia M Haile, Simon I Hay, Samuel

Hundessa, Hiroyasu Iso, Shubha Jayaram, Vidya Kadashetti, Ejaz Ahmad Khan, Moien AB Khan, Moawiah Mohammad Khatatbeh, Ali Kiadaliri, Min Seo Kim, Deborah R Kopansky-Giles, Jacek A Kopec, Kewal Krishan, Narinder Kumar, Thao Thi Thu Le, Ahmad Azam Malik, Lyn M March, Mohamed Kamal Mesregah, Tomislav Mestrovic, Arup Kumar Misra, Nouh Saad Mohamed, Ali H Mokdad, Kaveh Momenzadeh, Mohammad Ali Moni, Yousef Moradi, Satinath Mukhopadhyay, Christopher J L Murray, Sreenivas Narasimha Swamy, Van Thanh Nguyen, Robina Khan Niazi, Kanyin Liane Ong, Mayowa O Owolabi, Jagadish Rao Padubidri, Jay Patel, Shrikant Pawar, Paolo Pedersini, Salman Rawaf, Fatemeh Saheb Sharif-Askari, Amirhossein Sahebkar, Allen Seylani, Paramdeep Singh, Ranjan Solanki, Yonatan Solomon, Ker-Kan Tan, Nathan Y Tat, Theo Vos, Anthony D Woolf and Osama A Zitoun.

Managing the estimation or publications process

Marita Cross, Simon I Hay, Hailey Hagins, Ali H Mokdad, Christopher J L Murray, and Theo Vos.
